# Supplementary material for: Genomic instability in individuals with sex determination defects and germ cell cancer
Source: Cell Death Discov. 2023 May 23;9:173. doi: 10.1038/s41420-023-01470-6 (PMC10202957; doi:10.1038/s41420-023-01470-6)
Supplement: Supplementary file 1 — Extended data table 1 [file 41420_2023_1470_MOESM1_ESM.pdf]

Extended data table 1.
List of individuals with DSD.

	DSD groups	Age	Karyotype	Phenotype	Malignant Tumor (gonadal biopsy)	Gonadal tissue histology
	Swyer-GCT					
1	1	16	46, XY	Female	Dysgerminoma	CD117+, PLAP+
2	2	18	46, XY	Female	Seminoma/ Dysgerminoma	PAS+
3	3	17	46, XY (SRY mutation)	Female	Dysgerminoma	CD117+, D2-40+, OCT4+, PLAP+, SALL4+
4	4	32	46, XY	Female	Dysgerminoma, Gonadoblastoma	AFP+, β-HCG+
5	5	6	46, XY	Female	Dysgerminoma	KiA10+, AP +
	Swyer					
6	1	24	46, XY	Female	Not found	
7	2	16	46, XY	Female	Not found	
8	3	12	46, XY	Female	Not found	
9	4	10	46, XY	Female	Not found	
10	5	18	46, XY	Female	Not found	
11	6	16	46, XY	Female	Not found	
12	7	17	46, XY	Female	Not found	
	CAIS					
13	1	29	46, XY	Female	Not found	Negative for AFP, OCT4, SALL4, PLAP, S100, CD117, beta-HCG
14	2	45	46, XY	Female	Not found	Negative for S100, SALL4, AFP, C2-40, OCT4, PLAP, beta-HCG
15	3	14	46, XY	Female	Not found	
16	4	18	46, XY	Female	Not found	
17	5	9	46, XY	Female	Not found	
18	6	17	46, XY	Female	Not found	
19	7	17	46, XY	Female	Not found	
20	8	18	46, XY	Female	Not found	hyperplastic Leydig cells
21	9	32	46, XY	Female	Not found	Differentiated Sertoli-Leydig cells tumor, secondary Sertoli cells hyperplasia, no germ cells tumor, no malignancy<br>OCT4+, TSPY+, DDX3Y+
	CAIS-GCT			Female		
22	1		46, XY	Female	Dysgerminoma/ Gonadoblastoma	D2-40, OCT4, SALL4
	Turner					
23	1	14	45, X0	Female	No biopsy	
24	2	17	45, X0	Female	No biopsy	
25	3	10	45, X0	Female	No biopsy	
26	4	12	45, X0	Female	No biopsy	

27	5	16	45, X0	Female	No biopsy	
28	6	15	45, X0	Female	No biopsy	
29	7	11	45, X0	Female	No biopsy	
30	8	7	45, X0	Female	No biopsy	
31	9	6	45, X0	Female	No biopsy	
32	10	6	45, X0	Female	No biopsy	
33	11	12	45, X0	Female	No biopsy	
	Klinefelter					
34	1	34	XXY	Male	Not found	pronounced fibrosis
35	2	28	XXY	Male	Not found	
36	3	37	XXY	Male	Not found	
37	4	32	XXY	Male	Not found	pronounced fibrosis
38	5	37	XXY	Male	Not found	
39	6	36	XXY	Male	Not found	reactive Leydig cell hyperplasia
