# Supplementary material for: Genomic instability in individuals with sex determination defects and germ cell cancer
Source: Cell Death Discov. 2023 May 23;9:173. doi: 10.1038/s41420-023-01470-6 (PMC10202957; doi:10.1038/s41420-023-01470-6)
Supplement: Supplementary file 2 — Extended data table 2 [file 41420_2023_1470_MOESM2_ESM.pdf]

Extended data table 2.
List of control individuals.

Control group	Age	Karyotype	Phenotype
1	30	XY	Male
2	28	XY	Male
3	25	XY	Male
4	50	XY	Male
5	28	XY	Male
6	28	XX	Female
7	41	XX	Female
8	37	XX	Female
9	35	XX	Female
