# Supplementary material for: Genomic instability in individuals with sex determination defects and germ cell cancer
Source: Cell Death Discov. 2023 May 23;9:173. doi: 10.1038/s41420-023-01470-6 (PMC10202957; doi:10.1038/s41420-023-01470-6)
Supplement: Supplementary file 3 — Extended data table 3 [file 41420_2023_1470_MOESM3_ESM.pdf]

Extended data table 3.
List of TGCT-patients.

| TGCT | Age | Karyotype | Phenotype | Tumor |
|---|---|---|---|---|
| 1 | 38 | 46, XY | Male | Seminoma |
| 2 | 23 | 46, XY | Male | Seminoma;<br>Embryonal carcinoma;<br>Yolk sac tumor;<br>intratubular germ cell neoplasia unclassified |
| 3 | 55 | 46, XY | Male | Seminoma |
| 4 | 59 | 46, XY | Male | Embryonal carcinoma;<br>Teratoma |
| 5 | 34 | 46, XY | Male | Seminoma |
| 6 | 38 | 46, XY | Male | Seminoma, |
| 7 | 26 | 46, XY | Male | Seminoma;<br>Testicular intraepithelial neoplasia |
| 8 | 56 | 46, XY | Male | Seminoma |
| 9 | 45 | 46, XY | Male | Seminoma |
| 10 | 40 | 46, XY | Male | Seminoma |
| 11 | 46 | 46, XY | Male | Seminoma |
| 12 | 44 | 46, XY | Male | Seminoma |
| 13 | 32 | 46, XY | Male | Embryonic Carcinoma<br>Teratoma;<br>Chorionic Cancer;<br>Seminoma |
| 14 | 25 | 46, XY | Male | Seminoma;<br>Embryonal Carcinoma;<br>Yolk sac Tumor;<br>Chorionic Carcinoma |
