# Supplementary material for: Genomic instability in individuals with sex determination defects and germ cell cancer
Source: Cell Death Discov. 2023 May 23;9:173. doi: 10.1038/s41420-023-01470-6 (PMC10202957; doi:10.1038/s41420-023-01470-6)
Supplement: Supplementary file 4 — Extended data table 4 [file 41420_2023_1470_MOESM4_ESM.docx]

Extended data table 4.

List of antibodies.

| Antibody | Concentration/dilution used | Cat. No | Company |
| --- | --- | --- | --- |
| γH2AX | 1 mg/ml; 1/5000 | ab11174 | Abcam |
| H2AX | 1,078 mg/ml; 1/5000 | ab124781 | Abcam |
| α-actinin | 200 µg/ml; 1/30.000 | SC-17829 | Santa Cruz Biotechnology |
| dsDNA | 1/50 | SC-58749 | Santa Cruz Biotechnology |
| LC3 | 1/500 | #3868 | Cell Signaling |
| OCT4 | 1/300 | SC-5279 | Santa Cruz Biotechnology |
| P62 | 1/500 | #5114 | Cell Signaling |
| P53 | 1/500 | M7001 | Agilent (DAKO) |
| P21 | 1/500 | #2947 | Cell Signaling |
| STING | 1/50 | 13647S | Cell Signaling |
| p-ATM-S1981 | 1/500 | SC-47739 | Sant Cruz Biotechnology |
