# Supplementary material for: Genomic instability in individuals with sex determination defects and germ cell cancer
Source: Cell Death Discov. 2023 May 23;9:173. doi: 10.1038/s41420-023-01470-6 (PMC10202957; doi:10.1038/s41420-023-01470-6)
Supplement: Supplementary file 5 — Extended data table 5 [file 41420_2023_1470_MOESM5_ESM.pdf]

Extended data table 5.

List of TaqMan assays.

Target gene name	Reference
H2AFX	Hs00266783_s1
LC3	Hs00261291_m1
P62	Hs00177654_m1
IFNβ	Hs01077958_s1
ISG15	Hs00192713_m1
ISG56	Hs01675197_m1
IL6	Hs00174131_m1
LAMP2	Hs00903582_m1
CTSB	Hs00947439_m1
Endogenous control gene name	
TBP	Hs00427620_m1
HPRT	Hs99999909_m1
GAPDH	Hs02758991_g1
RPLPO	Hs99999902_m1
