# Supplementary material for: Genomic instability in individuals with sex determination defects and germ cell cancer
Source: Cell Death Discov. 2023 May 23;9:173. doi: 10.1038/s41420-023-01470-6 (PMC10202957; doi:10.1038/s41420-023-01470-6)
Supplement: Supplementary file 6 — Extended data figure 1 [file 41420_2023_1470_MOESM6_ESM.pdf]

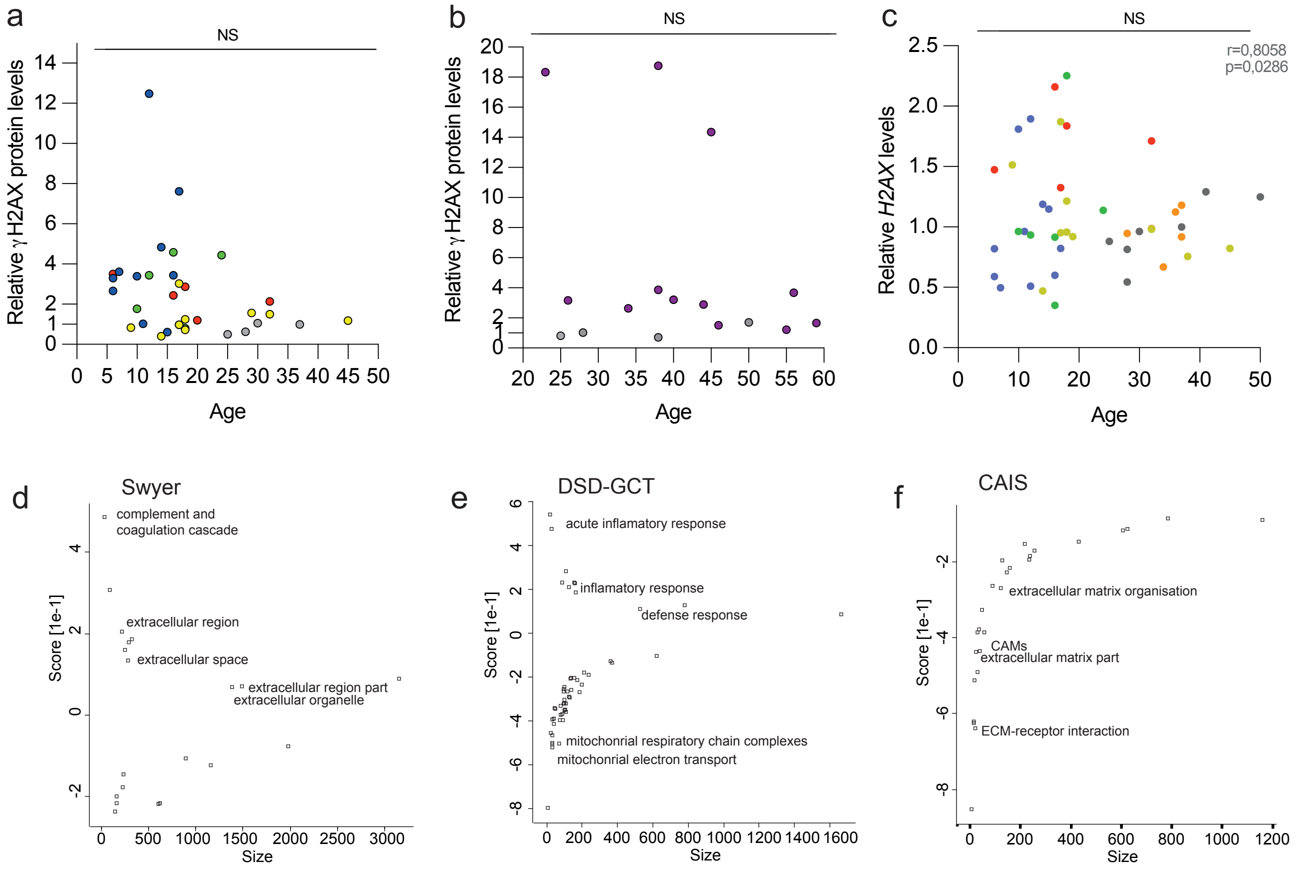

Extended data figure 1. Changes in DNA damage markers expression in leukocytes with age in DSD and TGCT-patients. γH2AX relative protein expression levels from DSD (a) and TGCT (b) samples groups correlated with age. (c) *H2AX* relative transcripts levels were plotted to correlate with age for DSD-patients. Pearson's correlation test was applied for statistical analysis and was positive for control group in (c). Colored dots correspond to different DSD-groups: Swyer-green, DSD-GCT-red, CAIS-yellow, Turner-blue; and control group - grey. Correlation coefficient and p values are indicated. All other cases didn't have statistically significant correlation. The deregulated pathways from whole proteome mass spectrometry analysis are represented from three Swyer (d) and three DSD-GCT (e) or CAIS (f) patients.
