# Supplementary material for: Genomic instability in individuals with sex determination defects and germ cell cancer
Source: Cell Death Discov. 2023 May 23;9:173. doi: 10.1038/s41420-023-01470-6 (PMC10202957; doi:10.1038/s41420-023-01470-6)
Supplement: Supplementary file 7 — Extended data figure 2 [file 41420_2023_1470_MOESM7_ESM.pdf]

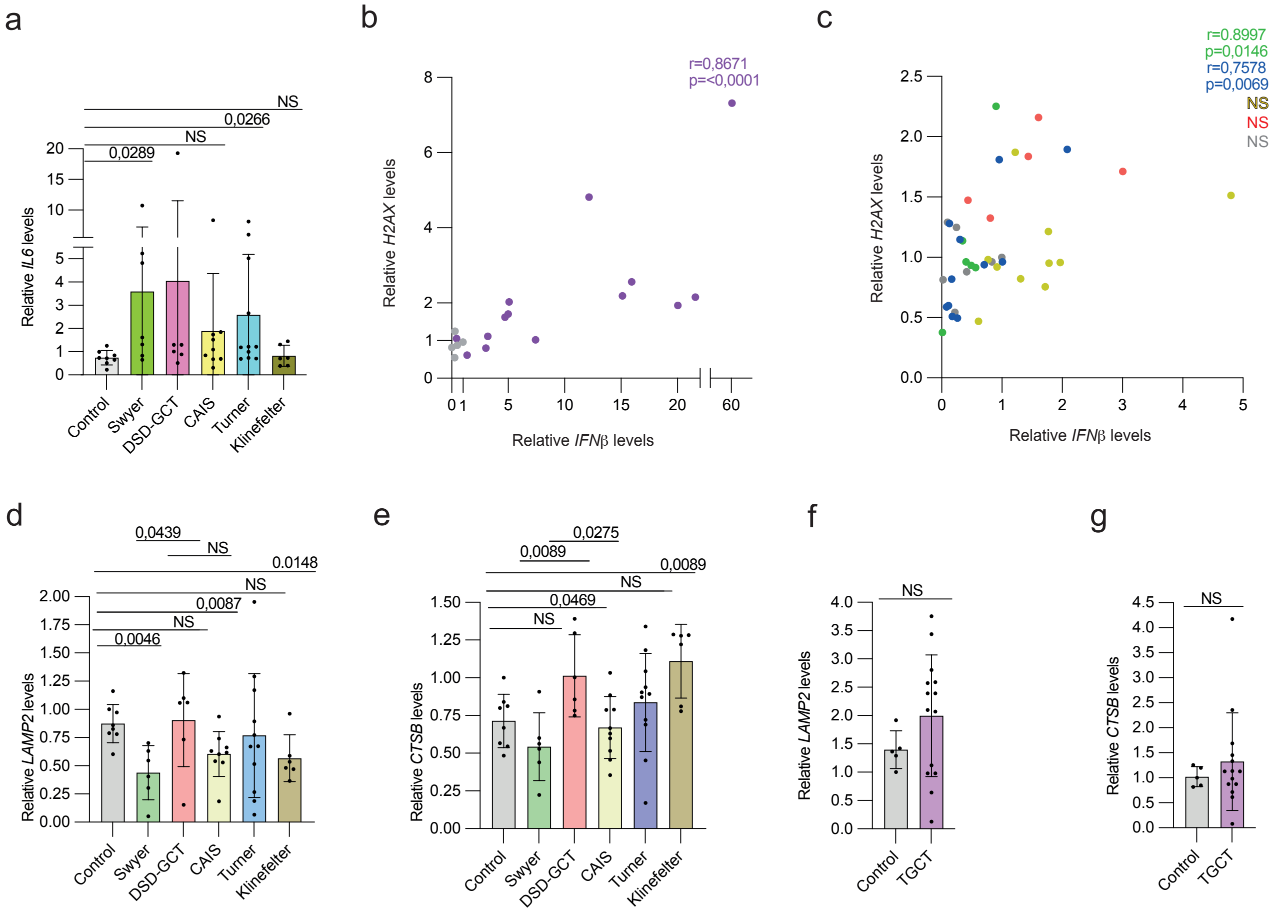

Extended data figure 2. DDR in leukocytes of DSD and TGCT-patients. qRT-PCR data of the NF-kB pathway target gene expression *IL6* in DSD-group (a). Correlation of DNA damage marker *H2AX* and innateimmune specific gene *IFN*β in TGCT (b) and DSD (c) groups (Swyer-green, DSD-GCT-red, CAIS-yellow, Turner-blue, control-grey). qRT-PCR data of lysosomal gene expression markers *LAMP2* and *CTSB* in DSD (d, e) and TGCT (f, g) samples. Pearson's correlation coefficients and p values after unpaired t-test are indicated.
