# Supplementary material for: Genomic instability in individuals with sex determination defects and germ cell cancer
Source: Cell Death Discov. 2023 May 23;9:173. doi: 10.1038/s41420-023-01470-6 (PMC10202957; doi:10.1038/s41420-023-01470-6)
Supplement: Supplementary file 8 — Extended data figure 3 [file 41420_2023_1470_MOESM8_ESM.pdf]

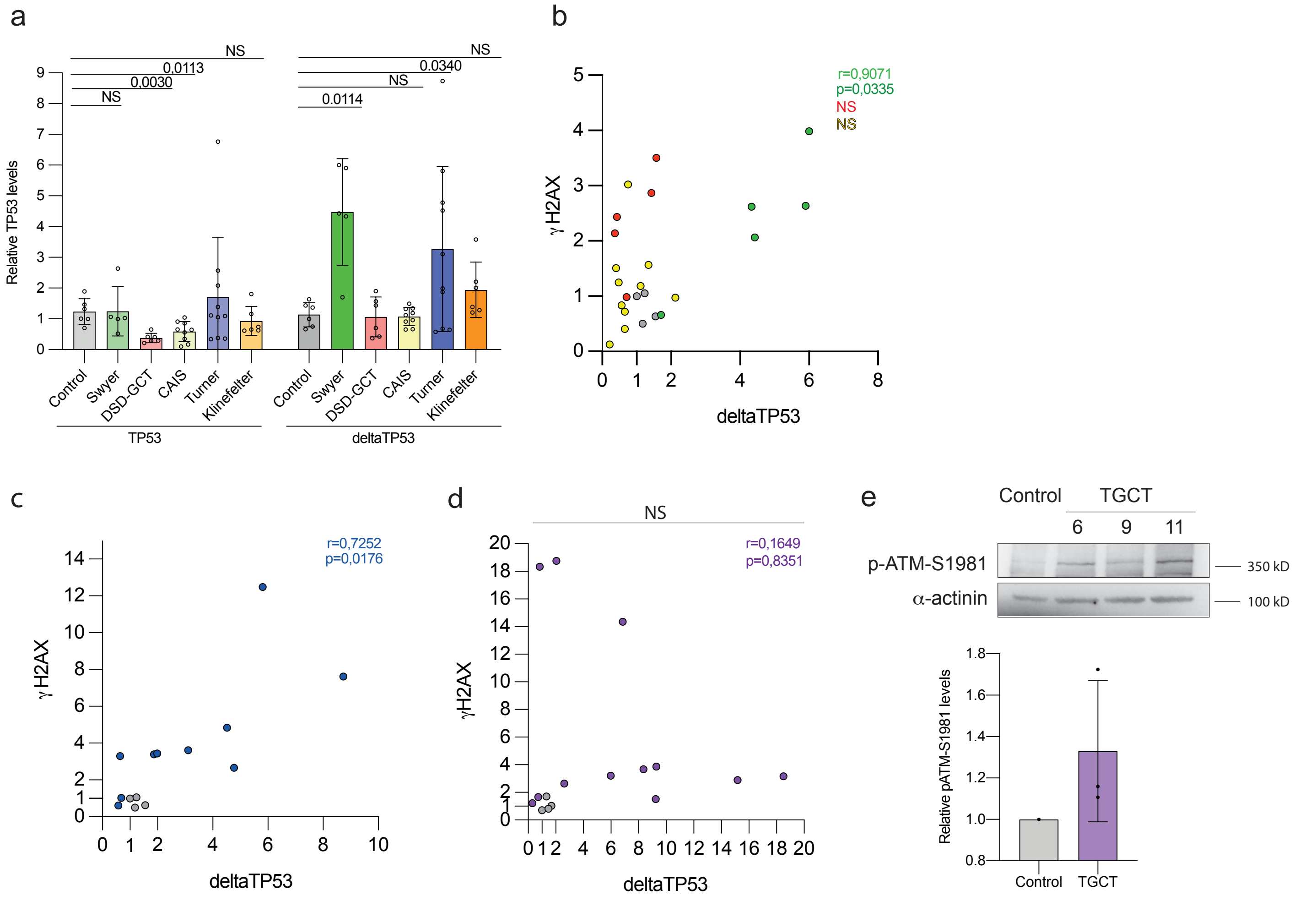

Extended data figure 3. Associated TP53 expression with DNA damage levels. (a) Quantification of TP53 protein levels relative to α-actinin from immunoblotings (Figure 4 a-e). Pearson's correlation was performed for DSD-samples (b, c). CAIS (yellow), DSD-GCT (red) and control (grey) groups didn't show significant correlation (b) as well as TGCT-group (d). Swyer (green) and Turner (blue)-samples showed significant correlation between γH2AX and TP53. Pearsons' correlation coefficients and p values from unpaired t test are indicated. e) Expression of the phosphorylated form in ATM in leukocytes from the periferal blood. Immunoblotting for p-ATM on serine resudue S1981, and a-actinin was used as the loading control. f) Quantification of the relative levels of p-ATM-S1981 from immunoblot (e).
