# Supplementary material for: Genomic instability in individuals with sex determination defects and germ cell cancer
Source: Cell Death Discov. 2023 May 23;9:173. doi: 10.1038/s41420-023-01470-6 (PMC10202957; doi:10.1038/s41420-023-01470-6)
Supplement: Supplementary file 9 — Extended data figure 4 [file 41420_2023_1470_MOESM9_ESM.pdf]

a

Exon 10

```
Swyer-GCT-1     ---CGGCGGGTTATCCTTCTTCCCCCCCCTGTTGCTGCAGATCGTGGGCGTGAGCGCTTC  57
Swyer-GCT-4     --AAGCCGGTATATCCTTCTTCCCCCCCCTGTTGCTGCAGATCGTGGGCGTGAGCGCTTC  58
TP53-control    -----------------------------------GCAGATCCGTGGGCGTGAGCGCTTC  25
Swyer-1         GGCTGGCAGGTTATCTTCGTTCCCCCCCCTGTTGCTGCAGATCGTGGGCGTGAGCGCTTC  60
Swyer-2         --TGCAGCCGGTTTCTTCGTTCCCCCCCCTGTTGCTGCAGATCGTGGGCGTGAGCGCTTC  58
                                                          ******************

Swyer-GCT-1     GAGATGTTCCGAGAGCTGAATGAGGCCTTGGAACTCAAGGATGCCCAGGCTGGGAAGGAG  117
Swyer-GCT-4     GAGATGTTCCGAGAGCTGAATGAGGCCTTGGAACTCAAGGATGCCCAGGCTGGGAAGGAG  118
TP53-control    GAGATGTTCCGAGAGCTGAATGAGGCCTTGGAACTCAAGGATGCCCAGGCTGGGAAGGAG  85
Swyer-1         GAGATGTTCCGAGAGCTGAATGAGGCCTTGGAACTCAAGGATGCCCAGGCTGGGAAGGAG  120
Swyer-2         GAGATGTTCCGAGAGCTGAATGAGGCCTTGGAACTCAAGGATGCCCAGGCTGGGAAGGAG  118
                ************************************************************

Swyer-GCT-1     CCAGGGGGGAGCAGGGCTCACTCCAGGTGAGTGACCTCAGCCCCTTCCTGGCCCTACTCC  177
Swyer-GCT-4     CCAGGGGGGAGCAGGGCTCACTCCAGGTGAGTGACCTCAGCCCCTTCCTGGCCCTACTCC  178
TP53-control    CCAGGGGGGAGCAGGGCTCACTCCAGGT--------------------------------  113
Swyer-1         CCAGGGGGGAGCAGGGCTCACTCCAGGTGAGTGACCTCAGCCCCTTCCTGGCCCTACTCC  180
Swyer-2         CCAGGGGGGAGCAGGGCTCACTCCAGGTGAGTGACCTCAGCCCCTTCCTGGCCCTACTCC  178
                ****************************
```

b

Exon 11

```
TP53-control ----------------------AGCCACCTGAAGTCCAAAAAGGGTCAGTCTACCTCCCG    38
Swyer-GCT-1  ----GCTCTCTCCTCCTTTGCTCCTCAGCCACTGAAGCCAAAAAGGGCAGCTACCTCCCG    56
Swyer-GCT-4  GTCCTCTCCCTCCTCTTTGCTCCTCAGCCTCCTGAAGCCAAAAAGGGCAGCTACCTCCCG    60
                                         *    *     ***  *     **********

TP53-control CCATAAAAAACTCATGTTCAAGACAGAAGGGCCTGACTCAGACTGACATTCTCCACTTCT    98
Swyer-GCT-1  CCATAAAAAACTCATGTTCAAGACAGAAGGGCCTGACTCAGACTGACATTCTCCACTTCT    116
Swyer-GCT-4  CCATAAAAAACTCATGTTCAAGACAGAAGGGCCTGACTCAGACTGACATTCTCCACTTCT    120
             ************************************************************

TP53-control TGTTCCCCACTGACAGCCTCCCACCCCCATCTCTCCCTCCCCTGCCATTTTGGGTTTTGG    158
Swyer-GCT-1  TGTTCCCCACTGACAGCCTCCCACCCCCATCTCTCCCTCCCCTGCCATTTTGGGTTTTGG    176
Swyer-GCT-4  TGTTCCCCACTGACAGCCTCCCACCCCCATCTCTCCCTCCCCTGCCATTTTGGGTTTTGG    180
             ************************************************************

TP53-control GTCTTTGAACCCTTGCTTGCAATAGGTGTGCGTCAGAAGCACCCAGGACTTCCATTTGCT    218
Swyer-GCT-1  GTCTTTGAACGCGTTGCAATGCAATGGGGTGTGCGTCAAA--------------------    216
Swyer-GCT-4  GTCTTTGAACCGCGTTGCATATGCAATGGTGTGCGTCAAA--------------------    220
             **********                 *     *   *
```

Extended Data Figure 4. DNA sequence alignment. Genomic DNA of two Swyer-GCT samples were sequenced on the regions encoding exon 10 (a) and exon 11 (b) of gene TP53.
