# Supplementary material for: Genomic instability in individuals with sex determination defects and germ cell cancer
Source: Cell Death Discov. 2023 May 23;9:173. doi: 10.1038/s41420-023-01470-6 (PMC10202957; doi:10.1038/s41420-023-01470-6)
Supplement: Supplementary file 14 — Supplementary dataset 5 [file 41420_2023_1470_MOESM14_ESM.pdf]

Supplementary dataset 5.

Exon 2 TP53 alignment to reference sequence.

```
Swyer-2
TP53-control

Query  23   CCGTCCGGGCTA-TGCCAGTGGGAGGAGCCGCAGT-AGATCCCTAGCGTCGAGCCCCCTC  80
            || ||||||  | ||||| | |||||||||||||| |||| |||||||||||||||||||
Sbjct  14   CCTTCCGGGTCACTGCCA-T-GGAGGAGCCGCAGTCAGAT-CCTAGCGTCGAGCCCCCTC  70

Query  81   TGAGTCAGGAAACATTTTCAGACCTATGGAAACTGTGAGTGGATCCATTGGAAGGGCAGG  140
            ||||||||||||||||||||||||||||||||||||||||||||||||||||||||||||
Sbjct  71   TGAGTCAGGAAACATTTTCAGACCTATGGAAACTGTGAGTGGATCCATTGGAAGGGCAGG  130

Query  141  CCCACCACCCCCGACCCCAACCCCAGCCCCCTAGCAGAGACCTGTGGGAAGCGAAAATTC  200
            |||||||||||| |||||||||||||||||||||||||||||||||||||||||||||||
Sbjct  131  CCCACCACCCCC-ACCCCAACCCCAGCCCCCTAGCAGAGACCTGTGGGAAGCGAAAATTC  189

Query  201  CATGGGACTGACTTTCTGCTCTTGTCTTTCAGACTTCCTGAAAACAACGTTCTGGT  256
            ||||||||||||||||||||||||||||||||||||||||||||||||||||||||
Sbjct  190  CATGGGACTGACTTTCTGCTCTTGTCTTTCAGACTTCCTGAAAACAACGTTCTGGT  245

Swyer-3
TP53-control

Query  19   CCATCCGGG-CCTTGCCATGGAGGAGCCGCAG-CAGAT-CTAGCG-CGAGCCCCCTCTGA  74
            || |||||| |  ||||||||||||||||||| ||||| |||||| ||||||||||||||
Sbjct  14   CCTTCCGGGTCACTGCCATGGAGGAGCCGCAGTCAGATCCTAGCGTCGAGCCCCCTCTGA  73

Query  75   G-CAGGAAACATTTTCAGACCTATGGAAACTGTGAGTGGATCCATTGGAAGGGCAGGCCC  133
            | ||||||||||||||||||||||||||||||||||||||||||||||||||||||||||
Sbjct  74   GTCAGGAAACATTTTCAGACCTATGGAAACTGTGAGTGGATCCATTGGAAGGGCAGGCCC  133

Query  134  ACCACCCCGACCCCAACCCCAGCCCCCTAGCAGAGACCTGTGGGAAGCGAAAAATTCCAT  193
            |||||||| |||||||||||||||||||||||||||||||||||||||| ||||||||||
Sbjct  134  ACCACCCCCACCCCAACCCCAGCCCCCTAGCAGAGACCTGTGGGAAGCG-AAAATTCCAT  192

Query  194  GGGACTGACTTTCTGCTCTTGTCTTTCAGACTTCCTGAAAACAACGTTCTGGT  246
            |||||||||||||||||||||||||||||||||||||||||||||||||||||
Sbjct  193  GGGACTGACTTTCTGCTCTTGTCTTTCAGACTTCCTGAAAACAACGTTCTGGT  245


Swyer-4
TP53-control

Query  23   TCCGGG-CA-TGCCATGGAGGAGCCGCAGT-AGAT-CTAGCGTCGAGCCCCCTCTGAGTC  78
            |||||| || |||||||||||||||||||| |||| ||||||||||||||||||||||||
Sbjct  17   TCCGGGTCACTGCCATGGAGGAGCCGCAGTCAGATCCTAGCGTCGAGCCCCCTCTGAGTC  76

Query  79   AGGAAACATTTTCAGACCTATGGAAACTGTGAGTGGATCCATTGGAAGGGCAGGCCCACC  138
            ||||||||||||||||||||||||||||||||||||||||||||||||||||||||||||
Sbjct  77   AGGAAACATTTTCAGACCTATGGAAACTGTGAGTGGATCCATTGGAAGGGCAGGCCCACC  136
```

```
Query  139  ACCCCGACCCCAACCCCAGCCCCCTAGCAGAGACCTGTGGGAAGCGAAAATTCCATGGGA  198
            ||||| ||||||||||||||||||||||||||||||||||||||||||||||||||||||
Sbjct  137  ACCCCCACCCCAACCCCAGCCCCCTAGCAGAGACCTGTGGGAAGCGAAAATTCCATGGGA  196

Query  199  CTGACTTTCTGCTCTTGTCTTTCAGACTTCCTGAAAACAACGTTCTGGT  247
            |||||||||||||||||||||||||||||||||||||||||||||||||
Sbjct  197  CTGACTTTCTGCTCTTGTCTTTCAGACTTCCTGAAAACAACGTTCTGGT  245
```

Swyer-5-ex2
TP53-control

```
Query  33   AGCAGCCCGCCTGCCT-CCGGG-CACTGCCAT-GA-GAGCCGCAGTCAG-TCCTAGCGTC  87
            ||||||| | |||||| ||||| ||||||||| || ||||||||||||| ||||||||||
Sbjct  1    AGCAGCCAGACTGCCTTCCGGGTCACTGCCATGGAGGAGCCGCAGTCAGATCCTAGCGTC  60

Query  88   GTGCCCCCTCTGAGTCAGGAAACATTTTCAGACCTATGGAA-CTGT-AGT-GATCCATTG  144
            | ||||||||||||||||||||||||||||||||||||||| |||| ||| |||||||||
Sbjct  61   GAGCCCCCTCTGAGTCAGGAAACATTTTCAGACCTATGGAAACTGTGAGTGGATCCATTG  120

Query  145  GAAGGG-A-GCCCACCACCCCCACCCCAACCCCAGCCCC-TAG-A-AG-CCTGTGG-AAG  197
            |||||| | |||||||||||||||||||||||||||||| ||| | || ||||||| |||
Sbjct  121  GAAGGGCAGGCCCACCACCCCCACCCCAACCCCAGCCCCCTAGCAGAGACCTGTGGGAAG  180

Query  198  -GAAA-TTCCATGGG-CTG-CTTTCTGCTCTTGTCTTTCAG-CTTCCT-AAAACAACGTT  251
             ||||  ||||||||| ||| ||||||||||||||||||||| |||||| |||||||||||
Sbjct  181  CGAAAATTCCATGGGACTGACTTTCTGCTCTTGTCTTTCAGACTTCCTGAAAACAACGTT  240

Query  252  CTGG  255
            ||||
Sbjct  241  CTGG  244
```

Swyer-7-ex2
TP53-control

```
Query  1    AGCAGCCAGACTGCCTTCCGGGTCACTGCCATGGAGGAGCCGCAGTCAGATCCTAGCGTC  60
            ||||||||||||||||||||||||||||||||||||||||||||||||||||||||||||
Sbjct  1    AGCAGCCAGACTGCCTTCCGGGTCACTGCCATGGAGGAGCCGCAGTCAGATCCTAGCGTC  60

Query  61   GAGCCCCCTCTGAGTCAGGAAACATTTTCAGACCTATGGAAACTGTGAGTGGATCCATTG  120
            ||||||||||||||||||||||||||||||||||||||||||||||||||||||||||||
Sbjct  61   GAGCCCCCTCTGAGTCAGGAAACATTTTCAGACCTATGGAAACTGTGAGTGGATCCATTG  120

Query  121  GAAGGGCAGGCCCACCACCCCGACCCCAACCCCAGCCCCCTAGCAGAGACCTGTGGGAAG  180
            ||||||||||||||||||||| ||||||||||||||||||||||||||||||||||||||
Sbjct  121  GAAGGGCAGGCCCACCACCCCCACCCCAACCCCAGCCCCCTAGCAGAGACCTGTGGGAAG  180

Query  181  CGAAAATTCCATGGGACTG-CTTTCTGCTCTTG-CTTTCAG-CTTCCTGAAAACAACGTT  237
            ||||||||||||||||||| ||||||||||||| ||||||| ||||||||||||||||||
Sbjct  181  CGAAAATTCCATGGGACTGACTTTCTGCTCTTGTCTTTCAGACTTCCTGAAAACAACGTT  240
```

```
Query  238  CTGG  241
            ||||
Sbjct  241  CTGG  244

Swyer-1-ex2       TATTATATGCCGATTCCCATTTTCCTCTCCAGCAGCCCGCCTGCTTCC--GGTCCCTCCC     58
TP53-control      ------------------------------AGCAGCCAGACTGCCTTCCGGGTCACTGCC     30
                                                  ******* * **** * *  **** ** **

Swyer-1-ex2       ATGAC---------GACCGAGTCTGTCTTAGGTCGCCACCCTCTAGTCAGAAACACTTTC     109
TP53-control      ATGGAGGAGCCGCAGTCAGATCCTAGCGTCG-AGCCCCCTCTGAGTCAGGAAACATTTTC     89
                  ***           * * **  **  * * *    ** * **       ****** ****

Swyer-1-ex2       ACACCATGGAACTGTAGGGG----GGCCTTGGAA-GGGAGGCGCGCCACCCCCGCCCTAA     164
TP53-control      AGACCTATGGAAACTGTGAGTGGATCCATTGGAAGGGCAGGCCCACCACCCCCACCCCAA     149
                  * ***   * *   *  * *      * ****** ** **** * ******** *** **

Swyer-1-ex2       CGCCCGCCCTTAAGA---GGATCTGCGGAAGCGAAATTCAC-------TGACTGCTTTCT     214
TP53-control      CCCCAGCCCCCTAGCAGAGACCTGTGGGAAGCGAAAATTCCATGGGACTGACTTTCTGCT     209
                  * ** ****   **    *       ********** *  *       *****   * **

Swyer-1-ex2       GTCCTGATGCAGCTTCCTAAAACACGCGGGACTGCCTGCGGTGCCTGAGCCTTTCGCCGT     274
TP53-control      CTTGTCTTTCAGACTTCCTGAAAACAACGTTCTGGT------------------------     245
                   *  *  * ***  * *   ** **   *  ***


Swyer6-ex2        TTCTATGCTGATCCCCCTTTTCCTCTTCAGAGCAGCTG-CTTCCGGGCACTGCCATGAGA     59
TP53-control      ----------AGCAGCCAGACTGCCTTCCGGGTCACTGCCATGGAGGAGCCGCAGTCA--     48
                            * *  **       **** * *   *** * *   **  * **  * *


Swyer6-ex2        GCCGAGTCAGTCCTAGCGCGGCCCCTCTAGTCAGGAACATTTTCAGCCATGGAACTGTA-     118
TP53-control      ---GATCCTAGCGTCGAGCCCCCTCTGAGTCAGGAAACATTTTCAGACCTATGGAAACTG     105
                     **  *   * * * **  ** **       * *********** * *

Swyer6-ex2        ----GTGGTCCATTGAAAGG--GAGCCCACCACCCCCACCCCAACCCCAGCCC------C     166
TP53-control      TGAGTGGATCCATTGGAAGGGCAGGCCCACCACCCCCACCCCAACCCCAGCCCCCTAGCA     165
                        * ******* ****    *****************************

Swyer6-ex2        TAGAAGCCTGTGGAAGCGAAATTCCATGGGCTGCTT--TCTGCTCTTTC---TTTCAGCT     221
TP53-control      GAGACCTGTGGGAAGCGAAAATTCCATGGGACTGACTTTCTGCTCTTGTCTTTCAGACTT     225
                   ***    ** * *    ************        *********     *   *  *

Swyer6-ex2        TCCTAAAACAACGTCTGGAAGGCAGGGAGGGCTGGGGCCTGGCGAAAGTATCGAGATAT      280
TP53-control      CCTGAAAACAACGTTCTGGT---------------------------------------      245
                   *  **********   *


DSD-GCT1-ex2
TP53-control

Query  22   TCCGGG-CCGTGCCATGGGAGGAGCCGCAGT-AGATCTTAGCGT-GAGCCCCCTCTGAGT  78
            |||||| |  |||||| |||||||||||||| ||||| |||||| |||||||||||||||
Sbjct  17   TCCGGGTCACTGCCAT-GGAGGAGCCGCAGTCAGATCCTAGCGTCGAGCCCCCTCTGAGT  75


Query  79   CAGGAAACATTTTCAGACCTATGGAAACTGTGAGTGGATCCATTGGAAGGGCAGGcccac  138
            ||||||||||||||||||||||||||||||||||||||||||||||||||||||||||||
Sbjct  76   CAGGAAACATTTTCAGACCTATGGAAACTGTGAGTGGATCCATTGGAAGGGCAGGCCCAC  135


Query  139  CACCCCCGACCCCAACCCCAGCCCCCTAGCAGAGACCTGTGGGAAGCGAAAATTCCATGG  198
            ||||||| ||||||||||||||||||||||||||||||||||||||||||||||||||||
Sbjct  136  CACCCCC-ACCCCAACCCCAGCCCCCTAGCAGAGACCTGTGGGAAGCGAAAATTCCATGG  194


Query  199  GACTGACTTTCTGCTCTTGTCTTTCAGACTTCCTGAAAACAACGTTCTGGT  249
            |||||||||||||||||||||||||||||||||||||||||||||||||||
Sbjct  195  GACTGACTTTCTGCTCTTGTCTTTCAGACTTCCTGAAAACAACGTTCTGGT  245
```

```
DSD-GCT2-ex2
TP53-control

Query  20   TCCGGG-CA-T-CCATGGAGGAGCCGCAG-CAGA-CCTAGCG-CGAGCCCCC-CTGAG-C  71
            |||||| || | ||||||||||||||||| |||| ||||||| ||||||||| ||||| |
Sbjct  17   TCCGGGTCACTGCCATGGAGGAGCCGCAGTCAGATCCTAGCGTCGAGCCCCCTCTGAGTC  76

Query  72   AGGAAACA-TTTCAGACCTATGGAAACTGTGAGTGGAT-CATTGGAAGGGCAGGCCCACC  129
            |||||||| ||||||||||||||||||||||||||||| |||||||||||||||||||||
Sbjct  77   AGGAAACATTTTCAGACCTATGGAAACTGTGAGTGGATCCATTGGAAGGGCAGGCCCACC  136

Query  130  ACCCCGACCCCAACCCCAGCCCCCTAGCAGAGACCTGTGGGAAGCGAAAATT-CATGGGA  188
            ||||| |||||||||||||||||||||||||||||||||||||||||||||| |||||||
Sbjct  137  ACCCCCACCCCAACCCCAGCCCCCTAGCAGAGACCTGTGGGAAGCGAAAATTCCATGGGA  196

Query  189  CTGACTTTCTGCTCTTGTCTTTCAGACTTCCTGAAAACAACGTTCTGGT  237
            |||||||||||||||||||||||||||||||||||||||||||||||||
Sbjct  197  CTGACTTTCTGCTCTTGTCTTTCAGACTTCCTGAAAACAACGTTCTGGT  245


DSD-GCT3-ex2
TP53-control

Query  19   GCC-TCCGGG-C-CTGCCATGGAGGAGCCGCAG-CAGAT-CTAGCG-CGAGCCCCCTCTG  72
            ||| |||||| | |||||||||||||||||||| ||||| |||||| |||||||||||||
Sbjct  13   GCCTTCCGGGTCACTGCCATGGAGGAGCCGCAGTCAGATCCTAGCGTCGAGCCCCCTCTG  72

Query  73   AGT-AGGAAACATTTTCAGACCTATGGAAACTGTGAGTGGATCCATTGGAAGGGCAGGcc  131
            ||| ||||||||||||||||||||||||||||||||||||||||||||||||||||||||
Sbjct  73   AGTCAGGAAACATTTTCAGACCTATGGAAACTGTGAGTGGATCCATTGGAAGGGCAGGCC  132

Query  132  CACCACCCCGACCCCAACCCCAGCCCCCTAGCAGAGACCTGTGGGAAGCGAAAATTCCAT  191
            ||||||||| ||||||||||||||||||||||||||||||||||||||||||||||||||
Sbjct  133  CACCACCCCCACCCCAACCCCAGCCCCCTAGCAGAGACCTGTGGGAAGCGAAAATTCCAT  192

Query  192  GGGACTGACTTTCTGCTCTTGTCTTTCAGACTTCCTGAAAACAACGTTCTGGT  244
            |||||||||||||||||||||||||||||||||||||||||||||||||||||
Sbjct  193  GGGACTGACTTTCTGCTCTTGTCTTTCAGACTTCCTGAAAACAACGTTCTGGT  245


DSD-GCT4-ex2
TP53-control

Query  51   AGCCGCAGT-AGATCCCTAGCG-CGAGCCCCCTCTGAG-CAGGAAAACATTTTCAAGACC  107
            ||||||||| |||| ||||||| ||||||||||||||| |||| |||||||||| |||||
Sbjct  38   AGCCGCAGTCAGAT-CCTAGCGTCGAGCCCCCTCTGAGTCAGG-AAACATTTTC-AGACC  94

Query  108  TATGGAAACTGTGAGTGGATCCATTGGAAGGGCAGGCCCACCACCCCCGACCCCAACCCC  167
            |||||||||||||||||||||||||||||||||||||||||||||||| |||||||||||
Sbjct  95   TATGGAAACTGTGAGTGGATCCATTGGAAGGGCAGGCCCACCACCCCC-ACCCCAACCCC  153
```

```
Query  168  agcccccTAGCAGAGACCTGTGGGAAGCGGAAAATTCCATGGGACTGACTTTCTGCTCTT  227
            |||||||||||||||||||||||||||| |||||||||||||||||||||||||||||||
Sbjct  154  AGCCCCCTAGCAGAGACCTGTGGGAAGC-GAAAATTCCATGGGACTGACTTTCTGCTCTT  212

Query  228  GTCTTTCAGACTTCCTGAAAACAACGTTCTGG  259
            ||||||||||||||||||||||||||||||||
Sbjct  213  GTCTTTCAGACTTCCTGAAAACAACGTTCTGG  244
```

DSD-GCT5-ex2
TP53-control

```
Query  47   GGAGAGAGCCGCAGT-AGATCTTAGCGTCGAGCCCCCTCTGAGTCAGGAAACATTTTCAG  105
            |||| |||||||||| ||||| ||||||||||||||||||||||||||||||||||||||
Sbjct  33   GGAG-GAGCCGCAGTCAGATCCTAGCGTCGAGCCCCCTCTGAGTCAGGAAACATTTTCAG  91

Query  106  ACCTATGGAAACTGTGAGTGGATCCATTGGAAGGGCAGGCCCACCACCCCCGACCCCAAC  165
            ||||||||||||||||||||||||||||||||||||||||||||||||||| ||||||||
Sbjct  92   ACCTATGGAAACTGTGAGTGGATCCATTGGAAGGGCAGGCCCACCACCCCC-ACCCCAAC  150

Query  166  CCCAGCCCCCTAGCAGAGACCTGTGGGAAGCGAAAATTCCATGGGACTGACTTTCTGCTC  225
            ||||||||||||||||||||||||||||||||||||||||||||||||||||||||||||
Sbjct  151  CCCAGCCCCCTAGCAGAGACCTGTGGGAAGCGAAAATTCCATGGGACTGACTTTCTGCTC  210

Query  226  TTGTCTTTCAGACTTCCTGAAAACAACGTTCTGGT  260
            |||||||||||||||||||||||||||||||||||
Sbjct  211  TTGTCTTTCAGACTTCCTGAAAACAACGTTCTGGT  245
```

CAIS-1-ex2
TP53-control-ex2

```
Query  58   CTGCCTTCCGGGTCACCTCGCCATGGAGGAACCGCAGTCAGATCCTAGCGTCGAGGCCCC  117
            |||||||||||||||| | ||||||||||| ||||||||||||||||||||||| |||||
Sbjct  11   CTGCCTTCCGGGTCAC-T-GCCATGGAGGAGCCGCAGTCAGATCCTAGCGTCGA-GCCCC  67

Query  118  CTCTGAGTCAGGAAACATTTTCAGACCTATGGAAACTGTGAGGTCAGATGCCACTTGGAA  177
            |||||||||||||||||||||||||||||||||||||||||| |  ||| ||| ||||||
Sbjct  68   CTCTGAGTCAGGAAACATTTTCAGACCTATGGAAACTGTGAG-TG-GAT-CCA-TTGGAA  123

Query  178  GGGCAGGCCACACCACACCCCACCCCAAACGACCAGCCCCCTAAGCAGAAGAACCTGCGG  237
            ||||||||| |||||| ||||||||||| |  ||||||||||| ||||| || |||| ||
Sbjct  124  GGGCAGGCC-CACCAC-CCCCACCCCAACC--CCAGCCCCCTA-GCAGA-GA-CCTGTGG  176

Query  238  GAAGGCGAAAATTCCACGGGACTGTCTTTCTCGCTCCTGTCTTTCAG-CTTCCTGAAAAC  296
            ||| |||||||||||| ||||||| |||||| |||| |||||||||| ||||||||||||
Sbjct  177  GAA-GCGAAAATTCCATGGGACTGACTTTCT-GCTCTTGTCTTTCAGACTTCCTGAAAAC  234

Query  297  ACAACGTGCTGG  308
            | | ||| ||||
Sbjct  235  A-A-CGTTCTGG  244
```

CAIS-4-ex2
TP53-control-ex2

```
Query  65    AGCAGCCAGACTGCCTTCCGGGTCACTGCCATGGAGGAGCCGCAGTCAGATCCTAGCGTC  124
             ||||||||||||||||||||||||||||||||||||||||||||||||||||||||||||
Sbjct  1     AGCAGCCAGACTGCCTTCCGGGTCACTGCCATGGAGGAGCCGCAGTCAGATCCTAGCGTC  60

Query  125   GAGCCCCCTCTGAGTCATGAAACATTTTCAG-CCTATGG-AACTGTGAGTGGATCCATTG  182
             ||||||||||||||||| ||||||||||||| ||||||| ||||||||||||||||||||
Sbjct  61    GAGCCCCCTCTGAGTCAGGAAACATTTTCAGACCTATGGAAACTGTGAGTGGATCCATTG  120

Query  183   GAAGGGCAGGCCCACCACCCCCACCCCAACCCCAGCCCCCTAGCAGAGACCTGTGGGAAA  242
             ||||||||||||||||||||||||||||||||||||||||||||||||||||||||| ||
Sbjct  121   GAAGGGCAGGCCCACCACCCCCACCCCAACCCCAGCCCCCTAGCAGAGACCTGTGGG-AA  179

Query  243   GCGAAAATTCCATGGG-CTG-CTTTCTGCTCTTGTCTTTCAG-CTTCCTGAAA-CAACGT  298
             |||||||||||||||| ||| ||||||||||||||||||||| |||||||||| ||||||
Sbjct  180   GCGAAAATTCCATGGGACTGACTTTCTGCTCTTGTCTTTCAGACTTCCTGAAAACAACGT  239

Query  299   -CTGG  302
              ||||
Sbjct  240   TCTGG  244
```

CAIS-5-ex2
TP53-control-ex2

```
Query  1     AGCAGCCAGACTGCCTTCCGGGTCACTGCCATGGAGGAGCCGCAGTCAGATCCTAGCGTC  60
             ||||||||||||||||||||||||||||||||||||||||||||||||||||||||||||
Sbjct  1     AGCAGCCAGACTGCCTTCCGGGTCACTGCCATGGAGGAGCCGCAGTCAGATCCTAGCGTC  60

Query  61    GAGCCCCCTCTGAGTCAGGAAACATTTTCAGACCTATGGAAACTGTGAGTGGATCCATTG  120
             ||||||||||||||||||||||||||||||||||||||||||||||||||||||||||||
Sbjct  61    GAGCCCCCTCTGAGTCAGGAAACATTTTCAGACCTATGGAAACTGTGAGTGGATCCATTG  120

Query  121   GAAGGGCAGGCCCACCACCCCCACCCCAACCCCAGCCCCCTAGCAGAGACCTGTGGGAAG  180
             ||||||||||||||||||||||||||||||||||||||||||||||||||||||||||||
Sbjct  121   GAAGGGCAGGCCCACCACCCCCACCCCAACCCCAGCCCCCTAGCAGAGACCTGTGGGAAG  180

Query  181   CGAAAATTCCATGGGACTGACTTTCTGCTCTTGTCTTTCAGACTTCCTGAAAACAACGTT  240
             ||||||||||||||||||||||||||||||||||||||||||||||||||||||||||||
Sbjct  181   CGAAAATTCCATGGGACTGACTTTCTGCTCTTGTCTTTCAGACTTCCTGAAAACAACGTT  240

Query  241   CTGGT  245
             |||||
Sbjct  241   CTGGT  245
```

CAIS-6-exon2
TP53-control

```
Query  36    AGCAGCCAGACTGCCTTCCGGGTCACTGCCATGGAGGAGCCGCAGTCAGATCCTAGCGTC  95
             ||||||||||||||||||||||||||||||||||||||||||||||||||||||||||||
Sbjct  1     AGCAGCCAGACTGCCTTCCGGGTCACTGCCATGGAGGAGCCGCAGTCAGATCCTAGCGTC  60
```

```
Query  96   GAGCCCCCTCTGAGTCAGGAAACATTTTCAGACCTATGGAAACTGTGAGTGGATCCATTG  155
            ||||||||||||||||||||||||||||||||||||||||||||||||||||||||||||
Sbjct  61   GAGCCCCCTCTGAGTCAGGAAACATTTTCAGACCTATGGAAACTGTGAGTGGATCCATTG  120

Query  156  GAAGGGCAGGCCCACCACCCCGACCCCAACCCCAGCCCCCTAGCAGAGACCTGTGGGAAG  215
            |||||||||||||||||||| |||||||||||||||||||||||||||||||||||||||
Sbjct  121  GAAGGGCAGGCCCACCACCCCCACCCCAACCCCAGCCCCCTAGCAGAGACCTGTGGGAAG  180

Query  216  CGAAAATTCCATGGGACTGACTTTCTGCTCTTGTCTTTCAG-CTTCCTGAAAACAACGTT  274
            ||||||||||||||||||||||||||||||||||||||||| ||||||||||||||||||
Sbjct  181  CGAAAATTCCATGGGACTGACTTTCTGCTCTTGTCTTTCAGACTTCCTGAAAACAACGTT  240

Query  275  CTGG  278
            ||||
Sbjct  241  CTGG  244
```

CAIS-8-exon2
TP53-control

```
Query  65   AGCAGCCAGACTGCCTTCCGGGTCACTGCCATGGAGGAGCCGCAGTCAGATCCTAGCGTC  124
            ||||||||||||||||||||||||||||||||||||||||||||||||||||||||||||
Sbjct  1    AGCAGCCAGACTGCCTTCCGGGTCACTGCCATGGAGGAGCCGCAGTCAGATCCTAGCGTC  60

Query  125  GAGCCCCCTCTGAGTCAGGAAACATTTTCAGACCTATGG-AACTGTGAGTGGATCCATTG  183
            ||||||||||||||||||||||||||||||||||||||| ||||||||||||||||||||
Sbjct  61   GAGCCCCCTCTGAGTCAGGAAACATTTTCAGACCTATGGAAACTGTGAGTGGATCCATTG  120

Query  184  GAAGGGCAGGCCCACCACCCCCACCCCAACCCCAGCCCCCTAGCAGAGACCTGTGGGAAA  243
            ||||||||||||||||||||||||||||||||||||||||||||||||||||||||| ||
Sbjct  121  GAAGGGCAGGCCCACCACCCCCACCCCAACCCCAGCCCCCTAGCAGAGACCTGTGGG-AA  179

Query  244  GCGAAAATTCCATGGG-CTG-CTTTCTGCTCTTGTCTTTCAG-CTTCCTGAAA-CAACGT  299
            |||||||||||||||| ||| ||||||||||||||||||||| |||||||||| ||||||
Sbjct  180  GCGAAAATTCCATGGGACTGACTTTCTGCTCTTGTCTTTCAGACTTCCTGAAAACAACGT  239

Query  300  -CTGG  303
             ||||
Sbjct  240  TCTGG  244
```

CAIS-9-exon2
TP53-control

```
Query  62   AGCAGCCAGACTGCCTTCCCGGGTCACTGCCATGGAGGAGCCGCAGTCAGATCCTAGCGT  121
            ||||||||||||||||| ||||||||||||||||||||||||||||||||||||||||||
Sbjct  1    AGCAGCCAGACTGCCTT-CCGGGTCACTGCCATGGAGGAGCCGCAGTCAGATCCTAGCGT  59

Query  122  CGAGCCCCCTCTGAGTCAGGAAACATTTTCAGACCTATGGAAACTGTGAGTGGATCCATT  181
            ||||||||||||||||||||||||||||||||||||||||||||||||||||||||||||
Sbjct  60   CGAGCCCCCTCTGAGTCAGGAAACATTTTCAGACCTATGGAAACTGTGAGTGGATCCATT  119
```

```
Query  182  GGAAAGGGCAGGCCCACCACCCCG-CCCCAACCCCAGCCCCCTAGCAGAG-CCTGTGG-A  238
            || ||||||||||||||||||||  |||||||||||||||||||||||| ||||||| |
Sbjct  120  GG-AAGGGCAGGCCCACCACCCCCACCCCAACCCCAGCCCCCTAGCAGAGACCTGTGGGA  178

Query  239  AGCGAAA-TTCCATGG-ACTG-CTTTCTGCTCTTGTCTTTCAG-CTTCCTGAAA-CAACG  293
            ||||||| |||||||| |||| |||||||||||||||||||| |||||||||| |||||
Sbjct  179  AGCGAAAATTCCATGGGACTGACTTTCTGCTCTTGTCTTTCAGACTTCCTGAAAACAACG  238

Query  294  T-CTGG  298
            | ||||
Sbjct  239  TTCTGG  244

CAIS-2-ex2           TTTTCTATGCTGGTCCCACTTTCCTCTTCAGAGCAGCTGCTTCCGGGCCCTCCATGGAGA    60
TP53-control-ex2     ----------AGCAGCCAGACTGCCTTCCGGGTCACTG------------CCATGGAGGA    38
                                *   ***   * *   * * *   **                *   *  **

CAIS-2-ex2           GCCGCAGTCAG------TCCTACGTCAGCCCCTCTAGTCAGAAACATTTTCACACCTATG    114
TP53-control-ex2     GCCGCAGTCAGATCCTAGCGTCGAGCCCCCTCTGAGTCAGGAAACATTTTCAGACCTATG    98
                     ***********       * *    *   ** **       ************ *******

CAIS-2-ex2           AAACTGTAGTGG---TCCATTGAA-GGGCAG-CCCACCACCCC-GCCCCAACCCCAG-CC    167
TP53-control-ex2     GAAACTGTGAGTGGATCCATTGGAAGGGCAGGCCCACCACCCCCACCCCAACCCCAGCCC    158
                      **      * *     ******* * ****** ***********  ************ **

CAIS-2-ex2           CCTA---GAAGCCTGTGGG---AGGAAATTCCATGG--GCTGCTTTCTGCTCTTGTCTTT    219
TP53-control-ex2     CCTAGCAGAGACCTGTGGGAAGCGAAAATTCCATGGGACTGACTTTCTGCTCTTGTCTTT    218
                     ****    **  ********     * ***********      ******************

CAIS-2-ex2           CAGCTTCCTAAAACAACGTCTGGAATGTCAGGGGTCCTGGGGCCTGGCTGGTCCGCCCCC    279
TP53-control-ex2     CAGACTTCCTGAAAACAACGTTCTGGT---------------------------------    245
                     ***   * *    ** *      *

CAIS-3-ex2           ATTCTCATGCTGGTCCCCACTTTTTTTTTGAGCAGCCAGCTGCTTCCGGGTCACTGCCAT    60
TP53-control-ex2     ----------------------------AGCAGCCAGACTGCCTTCCGGGTCACTGCCAT    32
                                                  *       *    ******************

CAIS-3-ex2           GAGAGCGCAGTC----AGTCCTAGCGTCA---GCCCCCTCTAGTCAGAAACATTTTCAGC    113
TP53-control-ex2     GGAGGAGCCGCAGTCAGATCCTAGCGTCGAGCCCCCTCTGAGTCAGGAAACATTTTCAGA    92
                     *   * ** *         **********      *** **       *************

CAIS-3-ex2           CTATGAAACT----GTAGTGATCCATTGAAGGGAGGCCACCA----CCCCGCCCCAACCC    165
TP53-control-ex2     CCTATGGAAACTGTGAGTGGATCCATTGGAAGGGCAGGCCCACCACCCCCACCCCAACCC    152
                     *       *       *     ********* * **       ***     **** *********

CAIS-3-ex2           CAGC-CCCTAGAA---GCCTGTGGA---AGGAAATTCCATGGGCTGCTTT---CTGTCTT    215
TP53-control-ex2     CAGCCCCCTAGCAGAGACCTGTGGGAAGCGAAAATTCCATGGGACTGACTTTCTGCTCTT    212
                     **** ****** *     *******      * ************       *      ****

CAIS-3-ex2           GTCTTTCAGCTTCC--TAAAACAACGTCTGGAATGGCAGGGTGGCTGGGGCCAGCGATCT    273
TP53-control-ex2     GTCTTTCAGACTTCCTGAAAACAACGTTCTGGT---------------------------    245
                     *********  * *   **********    *

CAIS-7-ex2           TATTCTCATGCTGATCCCCACTTTTTTTTTGCAGGAGGCAGCTGTCTTCCGGGTCACTG    60
TP53-control-ex2     --------------------------------AGCAGCCAGACTGCCTTCCGGGTCACTG    28
                                                                *** **************

CAIS-7-ex2           CCATGGCGAGCCG-CAG-TCTGTCCTAGCGTCGTGCCCCCTCTA--GTCAGAAACATTTT    116
TP53-control-ex2     CCATGGAGGAGCCGCAGTCAGATCCTAGCGTCGAGCCCCCTCTGAGTCAGGAAACATTTT    88
                     ****** *    *   ***     *********** *********        **********

CAIS-7-ex2           CAGCCTATGAAACTGTA----GTGATCCA-TTGAAGGGCAGGCCCACCACCCCGCCCCAA    171
TP53-control-ex2     CAGACCTATGGAAACTGTGAGTGGATCCATTGGAAGGGCAGGCCCACCACCCCCACCCCA    148
                     *** *       *   *        ****** * ********************  *** *

CAIS-7-ex2           CC-CCAGCC-CCTAG---AAGCCTGTGG--AA-GGAAATTCCATG--GGCTGCTTTCTGC    221
TP53-control-ex2     ACCCCAGCCCCCTAGCAGAGACCTGTGGGAAGCGAAAATTCCATGGGACTGACTTTCTGC    208
                      * ****** *****     *   *******   *   * **********       ********
```

```
CAIS-7-ex2            TCTTGTCTTTCAGCTTCCTA--AAACAACGTTCTGGAATGGCAGGGTGGGTGGGGCCGGC    279
TP53-control-ex2      TCTTGTCTTTCAGACTTCCTGAAAACAACGTTCTGGT-----------------------    245
                      *************  * *     **************
```

```
CAIS-GCT-exon2
TP53-control

Query  18   TCCGGGTC-CT-CCAT-GAGGAG-CG-AG-CAGA-CCTAGCG-CGAGCCCCC-CTGAG-C  67
            |||||||| || |||| |||||| || || |||| ||||||| ||||||||| ||||| |
Sbjct  17   TCCGGGTCACTGCCATGGAGGAGCCGCAGTCAGATCCTAGCGTCGAGCCCCCTCTGAGTC  76

Query  68   AGG-AACA-TTTCAGACCTATGGAAACTGTGAGTGGAT-CATTGGAAGGGCAGGCCCACC  124
            ||| |||| |||||||||||||||||||||||||||||| ||||||||||||||||||||
Sbjct  77   AGGAAACATTTTCAGACCTATGGAAACTGTGAGTGGATCCATTGGAAGGGCAGGCCCACC  136

Query  125  ACCCCCGACCCCAACCCCAGCCCCCTAGCAGAGACCTGTGGGAAGCGAAAATTCCATGGG  184
            |||||| |||||||||||||||||||||||||||||||||||||||||||||||||||||
Sbjct  137  ACCCCC-ACCCCAACCCCAGCCCCCTAGCAGAGACCTGTGGGAAGCGAAAATTCCATGGG  195

Query  185  ACTGACTTTCTGCTCTTGTCTTTCAGACTTCCTGAAAACAACGTTCTGGT  234
            ||||||||||||||||||||||||||||||||||||||||||||||||||
Sbjct  196  ACTGACTTTCTGCTCTTGTCTTTCAGACTTCCTGAAAACAACGTTCTGGT  245
```

```
Turner-1-exon2
TP53-control

Query  1    AGCAGCCAGACTGCCTTCCGGGTCACTGCCATGGAGGAGCCGCAGTCAGATCCTAGCGTC  60
            ||||||||||||||||||||||||||||||||||||||||||||||||||||||||||||
Sbjct  1    AGCAGCCAGACTGCCTTCCGGGTCACTGCCATGGAGGAGCCGCAGTCAGATCCTAGCGTC  60

Query  61   GAGCCCCCTCTGAGTCAGGAAACATTTTCAGACCTATGGAAACTGTGAGTGGATCCATTG  120
            ||||||||||||||||||||||||||||||||||||||||||||||||||||||||||||
Sbjct  61   GAGCCCCCTCTGAGTCAGGAAACATTTTCAGACCTATGGAAACTGTGAGTGGATCCATTG  120

Query  121  GAAGGGCAGGCCCACCACCCCCACCCCAACCCCAGCCCCCTAGCAGAGACCTGTGGGAAG  180
            ||||||||||||||||||||||||||||||||||||||||||||||||||||||||||||
Sbjct  121  GAAGGGCAGGCCCACCACCCCCACCCCAACCCCAGCCCCCTAGCAGAGACCTGTGGGAAG  180

Query  181  CGAAAATTCCATGGGACTGACTTTCTGCTCTTGTCTTTCAGACTTCCTGAAAACAACGTT  240
            ||||||||||||||||||||||||||||||||||||||||||||||||||||||||||||
Sbjct  181  CGAAAATTCCATGGGACTGACTTTCTGCTCTTGTCTTTCAGACTTCCTGAAAACAACGTT  240

Query  241  CTGGT  245
            |||||
Sbjct  241  CTGGT  245
```

```
Turner-2-exon2
TP53-control

Query  19   GCCGTCCGGGCTA-TGCCATGGAGGAGCCGCAG-CAGAT-CTAGCG-CGAGCCCCCTCTG  74
            ||| ||||||   | ||||||||||||||||||| ||||| |||||| |||||||||||||
Sbjct  13   GCCTTCCGGGTCACTGCCATGGAGGAGCCGCAGTCAGATCCTAGCGTCGAGCCCCCTCTG  72
```

```
Query  75   AGTCAGGAAACATTTTCAGACCTATGGAAACTGTGAGTGGATCCATTGGAAGGGCAGGCC  134
            ||||||||||||||||||||||||||||||||||||||||||||||||||||||||||||
Sbjct  73   AGTCAGGAAACATTTTCAGACCTATGGAAACTGTGAGTGGATCCATTGGAAGGGCAGGCC  132

Query  135  CACCACCCCGACCCCAACCCCAGCCCCCTAGCAGAGACCTGTGGGAAGCGAAAATTCCAT  194
            ||||||||| ||||||||||||||||||||||||||||||||||||||||||||||||||
Sbjct  133  CACCACCCCCACCCCAACCCCAGCCCCCTAGCAGAGACCTGTGGGAAGCGAAAATTCCAT  192

Query  195  GGGACTGACTTTCTGCTCTTGTCTTTCAGACTTCCTGAAAACAACGTTCTGGT  247
            |||||||||||||||||||||||||||||||||||||||||||||||||||||
Sbjct  193  GGGACTGACTTTCTGCTCTTGTCTTTCAGACTTCCTGAAAACAACGTTCTGGT  245
```

Turner-3-exon2
TP53-control

```
Query  17   CTTCCGTCCGGGCTA-TGCCATGGAGGAGCCGCAG-CAGATCCTAGCGT-GAGCCCCCTC  73
            || || ||||||  |  ||||||||||||||||||| ||||||||||||| ||||||||||
Sbjct  11   CTGCCTTCCGGGTCACTGCCATGGAGGAGCCGCAGTCAGATCCTAGCGTCGAGCCCCCTC  70

Query  74   TGAGTCAGGAAACATTTTCAGACCTATGGAAACTGTGAGTGGATCCATTGGAAGGGCAGG  133
            ||||||||||||||||||||||||||||||||||||||||||||||||||||||||||||
Sbjct  71   TGAGTCAGGAAACATTTTCAGACCTATGGAAACTGTGAGTGGATCCATTGGAAGGGCAGG  130

Query  134  CCCACCACCCCCACCCCAACCCCAGCCCCCTAGCAGAGACCTGTGGGAAGCGAAAATTCC  193
            ||||||||||||||||||||||||||||||||||||||||||||||||||||||||||||
Sbjct  131  CCCACCACCCCCACCCCAACCCCAGCCCCCTAGCAGAGACCTGTGGGAAGCGAAAATTCC  190

Query  194  ATGGGACTGACTTTCTGCTCTTGTCTTTCAGACTTCCTGAAAACAACGTTCTGGT  248
            |||||||||||||||||||||||||||||||||||||||||||||||||||||||
Sbjct  191  ATGGGACTGACTTTCTGCTCTTGTCTTTCAGACTTCCTGAAAACAACGTTCTGGT  245
```

Turner-4-exon2
TP53-control

```
Query  17   CTTCCGTCCGGGCTA-TGCCATGGAGGAGCCGCAG-CAGATCCTAGCGT-GAGCCCCCTC  73
            || || ||||||  |  ||||||||||||||||||| ||||||||||||| ||||||||||
Sbjct  11   CTGCCTTCCGGGTCACTGCCATGGAGGAGCCGCAGTCAGATCCTAGCGTCGAGCCCCCTC  70

Query  74   TGAGTCAGGAAACATTTTCAGACCTATGGAAACTGTGAGTGGATCCATTGGAAGGGCAGG  133
            ||||||||||||||||||||||||||||||||||||||||||||||||||||||||||||
Sbjct  71   TGAGTCAGGAAACATTTTCAGACCTATGGAAACTGTGAGTGGATCCATTGGAAGGGCAGG  130

Query  134  CCCACCACCCCCACCCCAACCCCAGCCCCCTAGCAGAGACCTGTGGGAAGCGAAAATTCC  193
            ||||||||||||||||||||||||||||||||||||||||||||||||||||||||||||
Sbjct  131  CCCACCACCCCCACCCCAACCCCAGCCCCCTAGCAGAGACCTGTGGGAAGCGAAAATTCC  190

Query  194  ATGGGACTGACTTTCTGCTCTTGTCTTTCAGACTTCCTGAAAACAACGTTCTGGT  248
            |||||||||||||||||||||||||||||||||||||||||||||||||||||||
Sbjct  191  ATGGGACTGACTTTCTGCTCTTGTCTTTCAGACTTCCTGAAAACAACGTTCTGGT  245
```

```
Turner-5-exon2
TP53-control

Query  1    AGCAGCCAGACTGCCTTCCGGGTCACTGCCATGGAGGAGCCGCAGTCAGATCCTAGCGTC  60
            ||||||||||||||||||||||||||||||||||||||||||||||||||||||||||||
Sbjct  1    AGCAGCCAGACTGCCTTCCGGGTCACTGCCATGGAGGAGCCGCAGTCAGATCCTAGCGTC  60

Query  61   GAGCCCCCTCTGAGTCAGGAAACATTTTCAGACCTATGGAAACTGTGAGTGGATCCATTG  120
            ||||||||||||||||||||||||||||||||||||||||||||||||||||||||||||
Sbjct  61   GAGCCCCCTCTGAGTCAGGAAACATTTTCAGACCTATGGAAACTGTGAGTGGATCCATTG  120

Query  121  GAAGGGCAGGCCCACCACCCCCACCCCAACCCCAGCCCCCTAGCAGAGACCTGTGGGAAG  180
            ||||||||||||||||||||||||||||||||||||||||||||||||||||||||||||
Sbjct  121  GAAGGGCAGGCCCACCACCCCCACCCCAACCCCAGCCCCCTAGCAGAGACCTGTGGGAAG  180

Query  181  CGAAAATTCCATGGGACTGACTTTCTGCTCTTGTCTTTCAGACTTCCTGAAAACAACGTT  240
            ||||||||||||||||||||||||||||||||||||||||||||||||||||||||||||
Sbjct  181  CGAAAATTCCATGGGACTGACTTTCTGCTCTTGTCTTTCAGACTTCCTGAAAACAACGTT  240

Query  241  CTGGT  245
            |||||
Sbjct  241  CTGGT  245

Turner-6-exon2
TP53-control

Query  23   CTTCCGGGGCCTAGTGCCATGGAGGAGCCGCAG-CAGATCCTAGCGTCGAGCCCCCTCTG  81
            ||||| ||| | | ||||||||||||||||||| ||||||||||||||||||||||||||
Sbjct  15   CTTCC-GGGTC-ACTGCCATGGAGGAGCCGCAGTCAGATCCTAGCGTCGAGCCCCCTCTG  72

Query  82   AGTCAGGAAACATTTTCAGACCTATGGAAACTGTGAGTGGATCCATTGGAAGGGCAGGCC  141
            ||||||||||||||||||||||||||||||||||||||||||||||||||||||||||||
Sbjct  73   AGTCAGGAAACATTTTCAGACCTATGGAAACTGTGAGTGGATCCATTGGAAGGGCAGGCC  132

Query  142  CACCACCCCCGACCCCAACCCCAGCCCCCTAGCAGAGACCTGTGGGAAGCGAAAATTCCA  201
            |||||||||| |||||||||||||||||||||||||||||||||||||||||||||||||
Sbjct  133  CACCACCCCC-ACCCCAACCCCAGCCCCCTAGCAGAGACCTGTGGGAAGCGAAAATTCCA  191

Query  202  TGGGACTGACTTTCTGCTCTTGTCTTTCAGACTTCCTGAAAACAACGTTCTGGT  255
            ||||||||||||||||||||||||||||||||||||||||||||||||||||||
Sbjct  192  TGGGACTGACTTTCTGCTCTTGTCTTTCAGACTTCCTGAAAACAACGTTCTGGT  245

Turner-7-exon2
TP53-control

Query  1    AGCAGCCAGACTGCCTTCCGGGTCACTGCCATGGAGGAGCCGCAGTCAGATCCTAGCGTC  60
            ||||||||||||||||||||||||||||||||||||||||||||||||||||||||||||
Sbjct  1    AGCAGCCAGACTGCCTTCCGGGTCACTGCCATGGAGGAGCCGCAGTCAGATCCTAGCGTC  60

Query  61   GAGCCCCCTCTGAGTCAGGAAACATTTTCAGACCTATGGAAACTGTGAGTGGATCCATTG  120
            ||||||||||||||||||||||||||||||||||||||||||||||||||||||||||||
Sbjct  61   GAGCCCCCTCTGAGTCAGGAAACATTTTCAGACCTATGGAAACTGTGAGTGGATCCATTG  120
```

```
Query  121   GAAGGGCAGGCCCACCACCCCCACCCCAACCCCAGCCCCCTAGCAGAGACCTGTGGGAAG  180
             ||||||||||||||||||||||||||||||||||||||||||||||||||||||||||||
Sbjct  121   GAAGGGCAGGCCCACCACCCCCACCCCAACCCCAGCCCCCTAGCAGAGACCTGTGGGAAG  180

Query  181   CGAAAATTCCATGGGACTGACTTTCTGCTCTTGTCTTTCAGACTTCCTGAAAACAACGTT  240
             ||||||||||||||||||||||||||||||||||||||||||||||||||||||||||||
Sbjct  181   CGAAAATTCCATGGGACTGACTTTCTGCTCTTGTCTTTCAGACTTCCTGAAAACAACGTT  240

Query  241   CTGGT  245
             |||||
Sbjct  241   CTGGT  245
```

Turner-8-exon2
TP53-control

```
Query  1     AGCAGCCAGACTGCCTTCCGGGTCACTGCCATGGAGGAGCCGCAGTCAGATCCTAGCGTC  60
             ||||||||||||||||||||||||||||||||||||||||||||||||||||||||||||
Sbjct  1     AGCAGCCAGACTGCCTTCCGGGTCACTGCCATGGAGGAGCCGCAGTCAGATCCTAGCGTC  60

Query  61    GAGCCCCCTCTGAGTCAGGAAACATTTTCAGACCTATGGAAACTGTGAGTGGATCCATTG  120
             ||||||||||||||||||||||||||||||||||||||||||||||||||||||||||||
Sbjct  61    GAGCCCCCTCTGAGTCAGGAAACATTTTCAGACCTATGGAAACTGTGAGTGGATCCATTG  120

Query  121   GAAGGGCAGGCCCACCACCCCCACCCCAACCCCAGCCCCCTAGCAGAGACCTGTGGGAAG  180
             ||||||||||||||||||||||||||||||||||||||||||||||||||||||||||||
Sbjct  121   GAAGGGCAGGCCCACCACCCCCACCCCAACCCCAGCCCCCTAGCAGAGACCTGTGGGAAG  180

Query  181   CGAAAATTCCATGGGACTGACTTTCTGCTCTTGTCTTTCAGACTTCCTGAAAACAACGTT  240
             ||||||||||||||||||||||||||||||||||||||||||||||||||||||||||||
Sbjct  181   CGAAAATTCCATGGGACTGACTTTCTGCTCTTGTCTTTCAGACTTCCTGAAAACAACGTT  240

Query  241   CTGGT  245
             |||||
Sbjct  241   CTGGT  245
```

Turner-9-exon2
TP53-control

```
Query  1     AGCAGCCAGACTGCCTTCCGGGTCACTGCCATGGAGGAGCCGCAGTCAGATCCTAGCGTC  60
             ||||||||||||||||||||||||||||||||||||||||||||||||||||||||||||
Sbjct  1     AGCAGCCAGACTGCCTTCCGGGTCACTGCCATGGAGGAGCCGCAGTCAGATCCTAGCGTC  60

Query  61    GAGCCCCCTCTGAGTCAGGAAACATTTTCAGACCTATGGAAACTGTGAGTGGATCCATTG  120
             ||||||||||||||||||||||||||||||||||||||||||||||||||||||||||||
Sbjct  61    GAGCCCCCTCTGAGTCAGGAAACATTTTCAGACCTATGGAAACTGTGAGTGGATCCATTG  120

Query  121   GAAGGGCAGGCCCACCACCCCCACCCCAACCCCAGCCCCCTAGCAGAGACCTGTGGGAAG  180
             ||||||||||||||||||||||||||||||||||||||||||||||||||||||||||||
Sbjct  121   GAAGGGCAGGCCCACCACCCCCACCCCAACCCCAGCCCCCTAGCAGAGACCTGTGGGAAG  180
```

```
Query  181  CGAAAATTCCATGGGACTGACTTTCTGCTCTTGTCTTTCAGACTTCCTGAAAACAACGTT  240
            ||||||||||||||||||||||||||||||||||||||||||||||||||||||||||||
Sbjct  181  CGAAAATTCCATGGGACTGACTTTCTGCTCTTGTCTTTCAGACTTCCTGAAAACAACGTT  240


Query  241  CTGGT  245
            |||||
Sbjct  241  CTGGT  245
```

Turner-10-exon2
TP53-control

```
Query  43   GCAGCCAGACTGGCTTCCGGGTCACTGCCATGGAGGAGCCGCAGTCAGATCCTAGCGTCG  102
            |||||||||||| |||||||||||||||||||||||||||||||||||||||||||||||
Sbjct  2    GCAGCCAGACTGCCTTCCGGGTCACTGCCATGGAGGAGCCGCAGTCAGATCCTAGCGTCG  61


Query  103  AGCCCCCTCTGAGTCAGGAAACATTTTCAGACCTATGGAAACTGTGAGTGGATCCATTGG  162
            ||||||||||||||||||||||||||||||||||||||||||||||||||||||||||||
Sbjct  62   AGCCCCCTCTGAGTCAGGAAACATTTTCAGACCTATGGAAACTGTGAGTGGATCCATTGG  121


Query  163  AAAGGGCAGGCCCACCACCCCGACCCCAACCCCAGCCCCCTAGCAGAGACCTGTGGGAAG  222
             |||||||||||||||||||| ||||||||||||||||||||||||||||||||||||||
Sbjct  122  -AAGGGCAGGCCCACCACCCCCACCCCAACCCCAGCCCCCTAGCAGAGACCTGTGGGAAG  180


Query  223  CGAAAATTCCATGGGACTG-CTTTCTGCTCTTGTCTTTCAG-CTTCCTGAAAACAACGTT  280
            ||||||||||||||||||| ||||||||||||||||||||| ||||||||||||||||||
Sbjct  181  CGAAAATTCCATGGGACTGACTTTCTGCTCTTGTCTTTCAGACTTCCTGAAAACAACGTT  240


Query  281  CTGG  284
            ||||
Sbjct  241  CTGG  244
```

Turner-11-exon2
TP53-control

```
Query  39   AGCAGGCCAAGACTGGCTTTCCCGGGTCACTGCCATGGAGGAGCCGCAGTCAGATTCCTA  98
            |||| ||| ||||| || || ||||||||||||||||||||||||||||||||| |||||
Sbjct  1    AGCA-GCC-AGACT-GCCTT-CCGGGTCACTGCCATGGAGGAGCCGCAGTCAGA-TCCTA  55


Query  99   GCGTCGAGCCCCCTCTGAGTCAGGAAACATTTTCAGACCTATGGAAACTGTGAGTGGATC  158
            ||||||||||||||||||||||||||||||||||||||||||||||||||||||||||||
Sbjct  56   GCGTCGAGCCCCCTCTGAGTCAGGAAACATTTTCAGACCTATGGAAACTGTGAGTGGATC  115


Query  159  CATTGGAAAGGGCAGGCCCACCACCCCGACCCCAACCCCAGCCCCCTAGCAGAGACCTGT  218
            |||||| |||||||||||||||||||| ||||||||||||||||||||||||||||||||
Sbjct  116  CATTGG-AAGGGCAGGCCCACCACCCCCACCCCAACCCCAGCCCCCTAGCAGAGACCTGT  174


Query  219  GGGAAGCGAAAATTCCATGGGACTG-CTTTCTGCTCTT-TCTTTCAG-CTTCCTGAAA-C  274
            ||||||||||||||||||||||||| |||||||||||| |||||||| |||||||||| |
Sbjct  175  GGGAAGCGAAAATTCCATGGGACTGACTTTCTGCTCTTGTCTTTCAGACTTCCTGAAAAC  234
```

```
Query  275   AACGT-CTGG   283
             ||||| ||||
Sbjct  235   AACGTTCTGG   244
```

Klinefelter-1-exon2
TP53-control

```
Query  1     AGCAGCCAGACTGCCTTCCGGGTCACTGCCATGGAGGAGCCGCAGTCAGATCCTAGCGTC  60
             ||||||||||||||||||||||||||||||||||||||||||||||||||||||||||||
Sbjct  1     AGCAGCCAGACTGCCTTCCGGGTCACTGCCATGGAGGAGCCGCAGTCAGATCCTAGCGTC  60

Query  61    GAGCCCCCTCTGAGTCAGGAAACATTTTCAGACCTATGGAAACTGTGAGTGGATCCATTG  120
             ||||||||||||||||||||||||||||||||||||||||||||||||||||||||||||
Sbjct  61    GAGCCCCCTCTGAGTCAGGAAACATTTTCAGACCTATGGAAACTGTGAGTGGATCCATTG  120

Query  121   GAAGGGCAGGCCCACCACCCCCACCCCAACCCCAGCCCCCTAGCAGAGACCTGTGGGAAG  180
             ||||||||||||||||||||||||||||||||||||||||||||||||||||||||||||
Sbjct  121   GAAGGGCAGGCCCACCACCCCCACCCCAACCCCAGCCCCCTAGCAGAGACCTGTGGGAAG  180

Query  181   CGAAAATTCCATGGGACTGACTTTCTGCTCTTGTCTTTCAGACTTCCTGAAAACAACGTT  240
             ||||||||||||||||||||||||||||||||||||||||||||||||||||||||||||
Sbjct  181   CGAAAATTCCATGGGACTGACTTTCTGCTCTTGTCTTTCAGACTTCCTGAAAACAACGTT  240

Query  241   CTGGT  245
             |||||
Sbjct  241   CTGGT  245
```

Klinefelter-2-exon2
TP53-control

```
Query  1     AGCAGCCAGACTGCCTTCCGGGTCACTGCCATGGAGGAGCCGCAGTCAGATCCTAGCGTC  60
             ||||||||||||||||||||||||||||||||||||||||||||||||||||||||||||
Sbjct  1     AGCAGCCAGACTGCCTTCCGGGTCACTGCCATGGAGGAGCCGCAGTCAGATCCTAGCGTC  60

Query  61    GAGCCCCCTCTGAGTCAGGAAACATTTTCAGACCTATGGAAACTGTGAGTGGATCCATTG  120
             ||||||||||||||||||||||||||||||||||||||||||||||||||||||||||||
Sbjct  61    GAGCCCCCTCTGAGTCAGGAAACATTTTCAGACCTATGGAAACTGTGAGTGGATCCATTG  120

Query  121   GAAGGGCAGGCCCACCACCCCCACCCCAACCCCAGCCCCCTAGCAGAGACCTGTGGGAAG  180
             ||||||||||||||||||||||||||||||||||||||||||||||||||||||||||||
Sbjct  121   GAAGGGCAGGCCCACCACCCCCACCCCAACCCCAGCCCCCTAGCAGAGACCTGTGGGAAG  180

Query  181   CGAAAATTCCATGGGACTGACTTTCTGCTCTTGTCTTTCAGACTTCCTGAAAACAACGTT  240
             ||||||||||||||||||||||||||||||||||||||||||||||||||||||||||||
Sbjct  181   CGAAAATTCCATGGGACTGACTTTCTGCTCTTGTCTTTCAGACTTCCTGAAAACAACGTT  240

Query  241   CTGGT  245
             |||||
Sbjct  241   CTGGT  245
```

Klinefelter-3-exon2
TP53-control

```
Query  24   TCCGGGCTA-TGCCATGGAGGAGCCGCAG-CAGAT-CTAGCG-CGAGCCCCCTCTGAG-C  78
            ||||||  | ||||||||||||||||||| ||||| |||||| ||||||||||||||| |
Sbjct  17   TCCGGGTCACTGCCATGGAGGAGCCGCAGTCAGATCCTAGCGTCGAGCCCCCTCTGAGTC  76

Query  79   AGGAAACATTTTCAGACCTATGGAAACTGTGAGTGGATCCATTGGAAGGGCAGGCCCACC  138
            ||||||||||||||||||||||||||||||||||||||||||||||||||||||||||||
Sbjct  77   AGGAAACATTTTCAGACCTATGGAAACTGTGAGTGGATCCATTGGAAGGGCAGGCCCACC  136

Query  139  ACCCCGACCCCAACCCCAGCCCCCTAGCAGAGACCTGTGGGAAGCGAAAATTCCATGGGA  198
            ||||| ||||||||||||||||||||||||||||||||||||||||||||||||||||||
Sbjct  137  ACCCCCACCCCAACCCCAGCCCCCTAGCAGAGACCTGTGGGAAGCGAAAATTCCATGGGA  196

Query  199  CTGACTTTCTGCTCTTGTCTTTCAGACTTCCTGAAAACAACGTTCTGGT  247
            |||||||||||||||||||||||||||||||||||||||||||||||||
Sbjct  197  CTGACTTTCTGCTCTTGTCTTTCAGACTTCCTGAAAACAACGTTCTGGT  245
```

Klinefelter-4-exon2
TP53-control

```
Query  1    AGCAGCCAGACTGCCTTCCGGGTCACTGCCATGGAGGAGCCGCAGTCAGATCCTAGCGTC  60
            ||||||||||||||||||||||||||||||||||||||||||||||||||||||||||||
Sbjct  1    AGCAGCCAGACTGCCTTCCGGGTCACTGCCATGGAGGAGCCGCAGTCAGATCCTAGCGTC  60

Query  61   GAGCCCCCTCTGAGTCAGGAAACATTTTCAGACCTATGGAAACTGTGAGTGGATCCATTG  120
            ||||||||||||||||||||||||||||||||||||||||||||||||||||||||||||
Sbjct  61   GAGCCCCCTCTGAGTCAGGAAACATTTTCAGACCTATGGAAACTGTGAGTGGATCCATTG  120

Query  121  GAAGGGCAGGCCCACCACCCCCACCCCAACCCCAGCCCCCTAGCAGAGACCTGTGGGAAG  180
            ||||||||||||||||||||||||||||||||||||||||||||||||||||||||||||
Sbjct  121  GAAGGGCAGGCCCACCACCCCCACCCCAACCCCAGCCCCCTAGCAGAGACCTGTGGGAAG  180

Query  181  CGAAAATTCCATGGGACTGACTTTCTGCTCTTGTCTTTCAGACTTCCTGAAAACAACGTT  240
            ||||||||||||||||||||||||||||||||||||||||||||||||||||||||||||
Sbjct  181  CGAAAATTCCATGGGACTGACTTTCTGCTCTTGTCTTTCAGACTTCCTGAAAACAACGTT  240

Query  241  CTGGT  245
            |||||
Sbjct  241  CTGGT  245
```

Klinefelter-5-exon2
TP53-control

```
Query  1    AGCAGCCAGACTGCCTTCCGGGTCACTGCCATGGAGGAGCCGCAGTCAGATCCTAGCGTC  60
            ||||||||||||||||||||||||||||||||||||||||||||||||||||||||||||
Sbjct  1    AGCAGCCAGACTGCCTTCCGGGTCACTGCCATGGAGGAGCCGCAGTCAGATCCTAGCGTC  60
```

```
Query  61   GAGCCCCCTCTGAGTCAGGAAACATTTTCAGACCTATGGAAACTGTGAGTGGATCCATTG  120
            ||||||||||||||||||||||||||||||||||||||||||||||||||||||||||||
Sbjct  61   GAGCCCCCTCTGAGTCAGGAAACATTTTCAGACCTATGGAAACTGTGAGTGGATCCATTG  120

Query  121  GAAGGGCAGGCCCACCACCCCCACCCCAACCCCAGCCCCCTAGCAGAGACCTGTGGGAAG  180
            ||||||||||||||||||||||||||||||||||||||||||||||||||||||||||||
Sbjct  121  GAAGGGCAGGCCCACCACCCCCACCCCAACCCCAGCCCCCTAGCAGAGACCTGTGGGAAG  180

Query  181  CGAAAATTCCATGGGACTGACTTTCTGCTCTTGTCTTTCAGACTTCCTGAAAACAACGTT  240
            ||||||||||||||||||||||||||||||||||||||||||||||||||||||||||||
Sbjct  181  CGAAAATTCCATGGGACTGACTTTCTGCTCTTGTCTTTCAGACTTCCTGAAAACAACGTT  240

Query  241  CTGGT  245
            |||||
Sbjct  241  CTGGT  245
```

```
Klinefelter-6-exon2
TP53-control
```

```
Query  39   AGCAGGCCAGGACCTGCCTTCCCGGGTCACTGCCATGGAGGAGCCGCAGTCAGATCCTAG  98
            |||| |||| || ||||||| |||||||||||||||||||||||||||||||||||||||
Sbjct  1    AGCA-GCCA-GA-CTGCCTT-CCGGGTCACTGCCATGGAGGAGCCGCAGTCAGATCCTAG  56

Query  99   CGTCGAGCCCCCTCTGAGTCAAGGAAACATTTTCAGACCTATGGAAACTGTGAGTGGATC  158
            |||||||||||||||||||| |||||||||||||||||||||||||||||||||||||||
Sbjct  57   CGTCGAGCCCCCTCTGAGTC-AGGAAACATTTTCAGACCTATGGAAACTGTGAGTGGATC  115

Query  159  CATTTGGAAGGGGCAGGCCCACCACCCCCACCCCAACCCCAGCCCCCTAGCAGAGACCTG  218
            |||| |||| ||||||||||||||||||||||||||||||||||||||||||||||||||
Sbjct  116  CATT-GGAA-GGGCAGGCCCACCACCCCCACCCCAACCCCAGCCCCCTAGCAGAGACCTG  173

Query  219  TGGGAAGCGAAAATTCCATGGGACTG-CTTTCTGCTCTTGTCTTTCAG-CTTCCTGAAA-  275
            |||||||||||||||||||||||||| ||||||||||||||||||||| |||||||||| 
Sbjct  174  TGGGAAGCGAAAATTCCATGGGACTGACTTTCTGCTCTTGTCTTTCAGACTTCCTGAAAA  233

Query  276  CAACGT-CTGG  285
            |||||| ||||
Sbjct  234  CAACGTTCTGG  244
```

Exon 4 TP53 alignment to reference sequence.

```
Swyer-1-exon4
TP53-control
```

```
Query  36   TTGCCGTCCCAAGCAATGGATGATTTGATGCTGTCCCCGGACGATATTGAACAATGGTTC  95
            ||||||||||||||||||||||||||||||||||||||||||||||||||||||||||||
Sbjct  9    TTGCCGTCCCAAGCAATGGATGATTTGATGCTGTCCCCGGACGATATTGAACAATGGTTC  68

Query  96   ACTGAAGACCCAGGTCCAGATGAAGCTCCCAGAATGCCAGAGGCTGCTCCCCGCGTGGCC  155
            |||||||||||||||||||||||||||||||||||||||||||||||||||| |||||||
Sbjct  69   ACTGAAGACCCAGGTCCAGATGAAGCTCCCAGAATGCCAGAGGCTGCTCCCCCCGTGGCC  128
```

```
Query  156  CCCGCACCAGCAGCTCCTACACCGGCGGCCCCTGCACCAGCCCCCTCCTGGCCCCTGTCA  215
            || |||||||||||||||||||||||||||||||||||||||||||||||||||||||||
Sbjct  129  CCTGCACCAGCAGCTCCTACACCGGCGGCCCCTGCACCAGCCCCCTCCTGGCCCCTGTCA  188

Query  216  TCTTCTGTCCCTTCCCAGAAAACCTACCAGGGCAGCTACGGTTTCCGTCTGGGCTTCTTG  275
            ||||||||||||||||||||||||||||||||||||||||||||||||||||||||||||
Sbjct  189  TCTTCTGTCCCTTCCCAGAAAACCTACCAGGGCAGCTACGGTTTCCGTCTGGGCTTCTTG  248

Query  276  CATTCTGGGACAGCCAAGTCTG-G-CTCGCACG  306
            |||||||||||||||||||||| | || |||||
Sbjct  249  CATTCTGGGACAGCCAAGTCTGTGACTTGCACG  281
```

Swyer-2-exon4
TP53-control

```
Query  34   CCTTGCCGTCCCAAGCAATGGATGATTTGATGCTGTCCCCGGACGATATTGAACAATGGT  93
            ||||||||||||||||||||||||||||||||||||||||||||||||||||||||||||
Sbjct  7    CCTTGCCGTCCCAAGCAATGGATGATTTGATGCTGTCCCCGGACGATATTGAACAATGGT  66

Query  94   TCACTGAAGACCCAGGTCCAGATGAAGCTCCCAGAATGCCAGAGGCTGCTCCCCCCGCGT  153
            |||||||||||||||||||||||||||||||||||||||||||||||||||||||  |||
Sbjct  67   TCACTGAAGACCCAGGTCCAGATGAAGCTCCCAGAATGCCAGAGGCTGCTCCCCC--CGT  124

Query  154  GGCCCCTGCACCAGCAGCTCCTACACCGGCGGCCCCTGCACCAGCCCCCTCCTGGCCCCT  213
            ||||||||||||||||||||||||||||||||||||||||||||||||||||||||||||
Sbjct  125  GGCCCCTGCACCAGCAGCTCCTACACCGGCGGCCCCTGCACCAGCCCCCTCCTGGCCCCT  184

Query  214  GTCATCTTCTGTCCCTTCCCAGAAAACCTACCAGGGCAGCTACGGTTTCCGTCTGGGCTT  273
            ||||||||||||||||||||||||||||||||||||||||||||||||||||||||||||
Sbjct  185  GTCATCTTCTGTCCCTTCCCAGAAAACCTACCAGGGCAGCTACGGTTTCCGTCTGGGCTT  244

Query  274  CTTGCATTCTGGGACAGCCAAGTCTG-G-CTTGCA  306
            |||||||||||||||||||||||||| | ||||||
Sbjct  245  CTTGCATTCTGGGACAGCCAAGTCTGTGACTTGCA  279
```

Swyer-3-exon4
TP53-control

```
Query  1    AGTCCCCCTTGCCGTCCCAAGCAATGGATGATTTGATGCTGTCCCCGG-CGATATTGAAC  59
            |||||||||||||||||||||||||||||||||||||||||||||||| |||||||||||
Sbjct  1    AGTCCCCCTTGCCGTCCCAAGCAATGGATGATTTGATGCTGTCCCCGGACGATATTGAAC  60

Query  60   AATGGTTCACTGAAGACCCAGGTCCAGATGAAGCTCCCAGAATGCCAGAGGCTGCTCCCC  119
            ||||||||||||||||||||||||||||||||||||||||||||||||||||||||||||
Sbjct  61   AATGGTTCACTGAAGACCCAGGTCCAGATGAAGCTCCCAGAATGCCAGAGGCTGCTCCCC  120

Query  120  GCGTGGCCCCTGCACCAGCAGCTCCTACACCGGCGGCCCCTGCACCAGCCCCCTCCTGGC  179
             |||||||||||||||||||||||||||||||||||||||||||||||||||||||||||
Sbjct  121  CCGTGGCCCCTGCACCAGCAGCTCCTACACCGGCGGCCCCTGCACCAGCCCCCTCCTGGC  180
```

```
Query  180   CCCTGTCATCTTCTGTCCCTTCCCAGAAAACCTACCAGGGCAGCTACGGTTTCCGTCTGG  239
             ||||||||||||||||||||||||||||||||||||||||||||||||||||||||||||
Sbjct  181   CCCTGTCATCTTCTGTCCCTTCCCAGAAAACCTACCAGGGCAGCTACGGTTTCCGTCTGG  240

Query  240   GCTTCTTGCATTCTGGGACAGCCAAGTCTGTGACTTTGCACGG  282
             |||||||||||||||||||||||||||||||||||| ||||||
Sbjct  241   GCTTCTTGCATTCTGGGACAGCCAAGTCTGTGACTT-GCACGG  282
```

Swyer-4-exon4
TP53-control

```
Query  34    CTTGCCGTCCCAAGCAATGGATGATTTGATGCTGTCCCCGGACGATATTGAACAATGGTT  93
             ||||||||||||||||||||||||||||||||||||||||||||||||||||||||||||
Sbjct  8     CTTGCCGTCCCAAGCAATGGATGATTTGATGCTGTCCCCGGACGATATTGAACAATGGTT  67

Query  94    CACTGAAGACCCAGGTCCAGATGAAGCTCCCAGAATGCCAGAGGCTGCTCCCCGCGTGGC  153
             ||||||||||||||||||||||||||||||||||||||||||||||||||||| ||||||
Sbjct  68    CACTGAAGACCCAGGTCCAGATGAAGCTCCCAGAATGCCAGAGGCTGCTCCCCCCGTGGC  127

Query  154   CCCCGCACCAGCAGCTCCTACACCGGCGGCCCCTGCACCAGCCCCCTCCTGGCCCCTGTC  213
             ||| ||||||||||||||||||||||||||||||||||||||||||||||||||||||||
Sbjct  128   CCCTGCACCAGCAGCTCCTACACCGGCGGCCCCTGCACCAGCCCCCTCCTGGCCCCTGTC  187

Query  214   ATCTTCTGTCCCTTCCCAGAAAACCTACCAGGGCAGCTACGGTTTCCGTCTGGGCTTCTT  273
             ||||||||||||||||||||||||||||||||||||||||||||||||||||||||||||
Sbjct  188   ATCTTCTGTCCCTTCCCAGAAAACCTACCAGGGCAGCTACGGTTTCCGTCTGGGCTTCTT  247

Query  274   GCATTCTGGGACAGCCAAGTCTG--ACTTGCA  303
             |||||||||||||||||||||||  |||||||
Sbjct  248   GCATTCTGGGACAGCCAAGTCTGTGACTTGCA  279
```

Swyer-5-exon4
TP53-control

```
Query  84    AGTCTCCCTCTTTGCCGTCCCAAGCAATGGATGATTTGATGCTGTCCCCGGACGATATTG  143
             |||| ||| | |||||||||||||||||||||||||||||||||||||||||||||||||
Sbjct  1     AGTC-CCC-C-TTGCCGTCCCAAGCAATGGATGATTTGATGCTGTCCCCGGACGATATTG  57

Query  144   AACAATGGTTCACTGAAGACCCAGGTCCAGATGAAGCTCCCAGAATGCCAGAGGCTGCTC  203
             ||||||||||||||||||||||||||||||||||||||||||||||||||||||||||||
Sbjct  58    AACAATGGTTCACTGAAGACCCAGGTCCAGATGAAGCTCCCAGAATGCCAGAGGCTGCTC  117

Query  204   CCCCCGCGTGGCCCCCGCACCAGCAGCTCCTACACCGGCGGCCCCTGCACCAGCCCCCTC  263
             ||||  ||||||||| ||||||||||||||||||||||||||||||||||||||||||||
Sbjct  118   CCCC--CGTGGCCCCTGCACCAGCAGCTCCTACACCGGCGGCCCCTGCACCAGCCCCCTC  175

Query  264   CTGGCCCCTGTCATCTTCTGTCCCTTCCCAGAAAACCTACCAGGGCAGCTACGGTTTCCG  323
             ||||||||||||||||||||||||||||||||||||||||||||||||||||||||||||
Sbjct  176   CTGGCCCCTGTCATCTTCTGTCCCTTCCCAGAAAACCTACCAGGGCAGCTACGGTTTCCG  235
```

```
Query  324   TCTGGGCTTCTTGCATTCTGGGACAGCCAAGTCTG-G-CTTGCAAGGT  369
             ||||||||||||||||||||||||||||||||||| | |||||| |||
Sbjct  236   TCTGGGCTTCTTGCATTCTGGGACAGCCAAGTCTGTGACTTGCACGGT  283
```

Swyer-6-exon4
TP53-control

```
Query  37    CTTGCCGTCCCAAGCAATGGATGATTTGATGCTGTCCCCGGACGATATTGAACAATGGTT  96
             ||||||||||||||||||||||||||||||||||||||||||||||||||||||||||||
Sbjct  8     CTTGCCGTCCCAAGCAATGGATGATTTGATGCTGTCCCCGGACGATATTGAACAATGGTT  67

Query  97    CACTGAAGACCCAGGTCCAGATGAAGCTCCCAGAATGCCAGAGGCTGCTCCCCCCGTGTG  156
             |||||||||||||||||||||||||||||||||||||||||||||||||||||||  |||
Sbjct  68    CACTGAAGACCCAGGTCCAGATGAAGCTCCCAGAATGCCAGAGGCTGCTCCCCCC--GTG  125

Query  157   GCCCCCGCACCAGCAGCTCCTACACCGGCGGCCCCTGCACCAGCCCCCTCCTGGCCCCTG  216
             ||||| ||||||||||||||||||||||||||||||||||||||||||||||||||||||
Sbjct  126   GCCCCTGCACCAGCAGCTCCTACACCGGCGGCCCCTGCACCAGCCCCCTCCTGGCCCCTG  185

Query  217   TCATCTTCTGTCCCTTCCCAGAAAACCTACCAGGGCAGCTACGGTTTCCGTCTGGGCTTC  276
             ||||||||||||||||||||||||||||||||||||||||||||||||||||||||||||
Sbjct  186   TCATCTTCTGTCCCTTCCCAGAAAACCTACCAGGGCAGCTACGGTTTCCGTCTGGGCTTC  245

Query  277   TTGCATTCTGGGACAGCCAAGTCTG-GACTTGCACG  311
             ||||||||||||||||||||||||| ||||||||||
Sbjct  246   TTGCATTCTGGGACAGCCAAGTCTGTGACTTGCACG  281
```

Swyer-7-exon4
TP53-control

```
Query  1     AGTCCCCCTTGCCGTCCCAAGCAATGGATGATTTGATGCTGTCCCCGGACGATATTGAAC  60
             ||||||||||||||||||||||||||||||||||||||||||||||||||||||||||||
Sbjct  1     AGTCCCCCTTGCCGTCCCAAGCAATGGATGATTTGATGCTGTCCCCGGACGATATTGAAC  60

Query  61    AATGGTTCACTGAAGACCCAGGTCCAGATGAAGCTCCCAGAATGCCAGAGGCTGCTCCCC  120
             ||||||||||||||||||||||||||||||||||||||||||||||||||||||||||||
Sbjct  61    AATGGTTCACTGAAGACCCAGGTCCAGATGAAGCTCCCAGAATGCCAGAGGCTGCTCCCC  120

Query  121   GCGTGGCCCCTGCACCAGCAGCTCCTACACCGGCGGCCCCTGCACCAGCCCCCTCCTGGC  180
              |||||||||||||||||||||||||||||||||||||||||||||||||||||||||||
Sbjct  121   CCGTGGCCCCTGCACCAGCAGCTCCTACACCGGCGGCCCCTGCACCAGCCCCCTCCTGGC  180

Query  181   CCCTGTCATCTTCTGTCCCTTCCCAGAAAACCTACCAGGGCAGCTACGGTTTCCGTCTGG  240
             ||||||||||||||||||||||||||||||||||||||||||||||||||||||||||||
Sbjct  181   CCCTGTCATCTTCTGTCCCTTCCCAGAAAACCTACCAGGGCAGCTACGGTTTCCGTCTGG  240

Query  241   GCTTCTTGCATTCTGGGACAGCCAAGTCTGTGACTTGCACGGT  283
             |||||||||||||||||||||||||||||||||||||||||||
Sbjct  241   GCTTCTTGCATTCTGGGACAGCCAAGTCTGTGACTTGCACGGT  283
```

DSD-GCT-1-exon4
TP53-control

```
Query  35   CCTTGCCGTCCCAAGCAATGGATGATTTTGATGCTGTCCCCGGACGATATTGAACCAATG  94
            ||||||||||||||||||||||||| |||||||||||||||||||||||||||| |||||
Sbjct  7    CCTTGCCGTCCCAAGCAATGGATGA-TTTGATGCTGTCCCCGGACGATATTGAA-CAATG  64

Query  95   GTTCACTGAAGACCCAGGTCCAGATGAAGCTCCCAGAATGCCAGAGGCTGCTCCCCCCGC  154
            |||||||||||||||||||||||||||||||||||||||||||||||||||||||||  |
Sbjct  65   GTTCACTGAAGACCCAGGTCCAGATGAAGCTCCCAGAATGCCAGAGGCTGCTCCCCC--C  122

Query  155  GTGGCCCCTGCACCAGCAGCTCCTACACCGGCGGCCCCTGCACCAGCCCCCTCCTGGCCC  214
            ||||||||||||||||||||||||||||||||||||||||||||||||||||||||||||
Sbjct  123  GTGGCCCCTGCACCAGCAGCTCCTACACCGGCGGCCCCTGCACCAGCCCCCTCCTGGCCC  182

Query  215  CTGTCATCTTCTGTCCCTTCCCAGAAAACCTACCAGGGCAGCTACGGTTTCCGTCTGGGC  274
            ||||||||||||||||||||||||||||||||||||||||||||||||||||||||||||
Sbjct  183  CTGTCATCTTCTGTCCCTTCCCAGAAAACCTACCAGGGCAGCTACGGTTTCCGTCTGGGC  242

Query  275  TTCTTGCATTCTGGGACAGCCAAGTCTG  302
            ||||||||||||||||||||||||||||
Sbjct  243  TTCTTGCATTCTGGGACAGCCAAGTCTG  270
```

DSD-GCT-2-exon4
TP53-control

```
Query  1    CCC-TGCCG-CTCATAGCAATGGATGATTTGTATGCTGT-CCCGG-CGATATTGAACAAT  56
            ||| ||||| | || |||||||||||||||| ||||||| ||||| ||||||||||||||
Sbjct  6    CCCTTGCCGTCCCA-AGCAATGGATGATTTG-ATGCTGTCCCCGGACGATATTGAACAAT  63

Query  57   GGTTCACTGAAGACCCAGGT-CAGATGAAGCTCCCAGAATGCCAGAGGCGTGCTCCCCCC  115
            |||||||||||||||||||| |||||||||||||||||||||||||||| ||||||||||
Sbjct  64   GGTTCACTGAAGACCCAGGTCCAGATGAAGCTCCCAGAATGCCAGAGGC-TGCTCCCCCC  122

Query  116  GTGGCCCCTGCACCAGCGAGCTCCTACACCGGCGGCCCCTGCACCAGCCCCCTCCTGGGC  175
            ||||||||||||||||| |||||||||||||||||||||||||||||||||||||| |||
Sbjct  123  GTGGCCCCTGCACCAGC-AGCTCCTACACCGGCGGCCCCTGCACCAGCCCCCTCCT-GGC  180

Query  176  CCCTGTCATCTTTCTGTCCCTTTCCCAGAAAAACCTACCAGGGCAGCTACGGGTTTCCGT  235
            |||||||||||| ||||||||| ||||| ||||||||||||||||||||||| |||||||
Sbjct  181  CCCTGTCATCTT-CTGTCCCTT-CCCAG-AAAACCTACCAGGGCAGCTACGG-TTTCCGT  236

Query  236  CCTGGGCTTTCTTTGCATTCGTGGGGAACAGCCAAAGTCTTGTGACTTTGCACGG  290
            | ||||||| ||| |||||| |||| | ||||||| |||| ||||||| ||||||
Sbjct  237  C-TGGGCTT-CTT-GCATTC-TGGG-A-CAGCCAA-GTCT-GTGACTT-GCACGG  282
```

```
DSD-GCT-4-exon4
TP53-control

Query  33   CTTGCCGTCCCCAAGCAATGGATTGATTTGATGCTGTCCCCGGACGATATTGAACAATGG  92
            |||||||| ||||||||||||| |||||||||||||||||||||||||||||||||||||
Sbjct  8    CTTGCCGT-CCCAAGCAATGGA-TGATTTGATGCTGTCCCCGGACGATATTGAACAATGG  65

Query  93   TTCACTGAAGACCCAGGTCCAGATGAAGCTCCCAGAATGCCAGAGGCTGCTCCCCCCGCG  152
            ||||||||||||||||||||||||||||||||||||||||||||||||||||||||  ||
Sbjct  66   TTCACTGAAGACCCAGGTCCAGATGAAGCTCCCAGAATGCCAGAGGCTGCTCCCCC--CG  123

Query  153  TGGCCCCTGCACCAGCAGCTCCTACACCGGCGGCCCCTGCACCAGCCCCCTCCTGGCCCC  212
            ||||||||||||||||||||||||||||||||||||||||||||||||||||||||||||
Sbjct  124  TGGCCCCTGCACCAGCAGCTCCTACACCGGCGGCCCCTGCACCAGCCCCCTCCTGGCCCC  183

Query  213  TGTCATCTTCTGTCCCTTCCCAGAAAACCTACCAGGGCAGCTACGGTTTCCGTCTGGGCT  272
            ||||||||||||||||||||||||||||||||||||||||||||||||||||||||||||
Sbjct  184  TGTCATCTTCTGTCCCTTCCCAGAAAACCTACCAGGGCAGCTACGGTTTCCGTCTGGGCT  243

Query  273  TCTTGCATTCTGGGACAAGCCAA  295
            ||||||||||||||||| |||||
Sbjct  244  TCTTGCATTCTGGGACA-GCCAA  265

DSD-GCT-5-exon4
TP53-control

Query  5    TGCCG-CCTTAGCGATGGATGATTTGATGCTGTGTCCCGGACGATATTGAACAATGGTTC  63
            ||||| ||  ||| |||||||||||||||||||  |||||||||||||||||||||||||
Sbjct  10   TGCCGTCCCAAGCAATGGATGATTTGATGCTGT-CCCCGGACGATATTGAACAATGGTTC  68

Query  64   ACTGAAGACCCAGGTCCAGATGAAGCTCCCAGAATGCCAGAGGCGTGCTCCCCCCGTGGC  123
            |||||||||||||||||||||||||||||||||||||||||||| |||||||||||||||
Sbjct  69   ACTGAAGACCCAGGTCCAGATGAAGCTCCCAGAATGCCAGAGGC-TGCTCCCCCCGTGGC  127

Query  124  CCCTGCACCAGCGAGCTCCTACACCGGCGGCCCCTGCACCAGCCCCCTCCTGGCCCCTGT  183
            |||||||||||| |||||||||||||||||||||||||||||||||||||||||||||||
Sbjct  128  CCCTGCACCAGC-AGCTCCTACACCGGCGGCCCCTGCACCAGCCCCCTCCTGGCCCCTGT  186

Query  184  CATCTTCTGTCCCTTCCCAGAAAACCTACCAGGGCAGCTACGGTTTCCGTCTGGGCTTCT  243
            ||||||||||||||||||||||||||||||||||||||||||||||||||||||||||||
Sbjct  187  CATCTTCTGTCCCTTCCCAGAAAACCTACCAGGGCAGCTACGGTTTCCGTCTGGGCTTCT  246

Query  244  TGCATTCTGGGACAGCCAAGTCTGTGACTTGCACGGT  280
            |||||||||||||||||||||||||||||||||||||
Sbjct  247  TGCATTCTGGGACAGCCAAGTCTGTGACTTGCACGGT  283
```

```
CAIS-8-exon4
TP53-control

Query  17   GATGATTTGATGCTG-CCCCGG-CGATATTG-ACAATGGTCTACTGAAGACCCAGGTCCA  73
            ||||||||||||||| |||||| |||||||| ||||||||  ||||||||||||||||||
Sbjct  27   GATGATTTGATGCTGTCCCCGGACGATATTGAACAATGGTTCACTGAAGACCCAGGTCCA  86

Query  74   GATGAAGCT-CCAGAATGCCAGAGG-Tgcctccccccgt-g-ccct-caccagca-ct-c  126
            ||||||||| ||||||||||||||| || |||||||||| | |||| |||||||| || |
Sbjct  87   GATGAAGCTCCCAGAATGCCAGAGGCTG-CTCCCCCCGTGGCCCCTGCACCAGCAGCTCC  145

Query  127  t-c-ccgccgccccctgccccagccccctcctggcccctg-catcttctgcccctTCCaa  183
            | | ||| || ||||||| ||||||||||||||||||||| ||||||||| ||||||| |
Sbjct  146  TACACCGGCGGCCCCTGCACCAGCCCCCTCCTGGCCCCTGTCATCTTCTGTCCCTTCCCA  205

Query  184  aaaaaCCAACAAGGGCAGCTACGGTTTCCGTCTTGGCCTTCTTGCA-TCTGGGACAGCCA  242
             |||||| || |||||||||||||||||||||| ||| |||||||| |||||||||||||
Sbjct  206  GAAAACCTACCAGGGCAGCTACGGTTTCCGTCTGGGC-TTCTTGCATTCTGGGACAGCCA  264

Query  243  AGTCTGT  249
            |||||||
Sbjct  265  AGTCTGT  271

CAIS-9-exon4
TP53-control

CAIS-9-exon4      AGTCCCTCTTGCCGTCCAACATGATGGATGATTTGATGCTGTCCCCGGCGATA-TTGAAC     59
TP53-control      AGTCCCCCTTGCCGTCCCAAGCAATGGATGATTTGATGCTGTCCCCGGACGATATTGAAC     60
                  ****** ********** *    *************************      ******

CAIS-9-exon4      AATGGTC-ACTGA-AGACCCAGGCCAGATGAAGCTCCCAGAATGCCAGAGCGTGCCCCCC     117
TP53-control      AATGGTTCACTGAAGACCCAGGTCCAGATGAAGCTCCCAGAATGCCAGAGGCTGCTCCCC     120
                  ******  *****    **  * ***************************  *** ****

CAIS-9-exon4      GCGTGGCCCTGCAC-CAGCGGCT-CTACACCGGCGG-CCCCTCACCAGCCCCCTCCTGGG     174
TP53-control      CCGTGGCCCCTGCACCAGCAGCTCCTACACCGGCGGCCCCTGCACCAGCCCCCTCCTGGC     180
                   ********      **** *** ************ ***  *****************

CAIS-9-exon4      CCCCGGCACCTTCTGCCCCTCCAGA--AAACCTACCA-GGGAGCTACGGTTTCCGCTTGG     231
TP53-control      CCCTGTCATCTTCTGTCCCTTCCCAGAAAACCTACCAGGGCAGCTACGGTTTCCGTCTGG     240
                  *** * ** ****** **** *  *  ********** ** **************  ***

CAIS-9-exon4      C-TTCTTGATTGGGGACACC----AAGCTGTGAGTGGACGGGCAGTGGCCTAGGGCTGCT     286
TP53-control      GCTTCTTGCATTCTGGGACAGCCAAGTCTGTGACTTGCACGGT-----------------     283
                    ******  *   *  **     *  ****** * *   **

CAIS-GCT-exon4
TP53-control

CAIS-GCT-exon4    -----------CCCTCGCGCTTAAGTATGGATGATTTGATCTGCCCCGGCGATATTGAAC     49
TP53-control      AGTCCCCCTTGCCGTCCCAAGCAATGGATGATTTGATGCTGTCCCCGGACGATATTGAAC     60
                             ** ** *    **     ***    ** * * *** * ***********

CAIS-GCT-exon4    AATGGTCACTG-AAGACCCAG-GCCAGATGAAGCTCCAGAATCCAGAGCGC---------     98
TP53-control      AATGGTTCACTGAAGACCCAGGTCCAGATGAAGCTCCCAGAATGCCAGAGGCTGCTCCCC     120
                  ******      *********  **************   *     ** *

CAIS-GCT-exon4    CCCCCCCCGTGCCCTCACCACGCCTCTCACCGGCGGCCCCTGCCCCAGCCCCCTCCTGGC     158
TP53-control      CCGTGGCCCCTGCACCAGCAGCTCCTACACCGGCGGCCCCTGCACCAGCCCCCTCCTGGC     180
                  **    **    *  ** **   *   **************** ****************

CAIS-GCT-exon4    CCCTGC--ATTTTTGCCCCTTCCCAGAAAACCAACAAGGGCAGCTACGGTTTCCGCCTGG     216
TP53-control      CCCTGTCATCTTCTGTCCCTTCCCAGAAAACCTACCAGGGCAGCTACGGTTTCCGTCTGG     240
                  *****     ** ** **************** ** ******************* ****
```

```
CAIS-GCT-exon4      CTT--CTGCATTCTGGGACAGCCAAGCCTGTTACTTGGACGGGCAAGTGCCTGAGGGCTG      274
TP53-control        GCTTCTTGCATTCTGGGACAGCCAAGTCTGTGACTTGCACGGT-----------------      283
                      *   ******************** **** ***** ****
```

```
Turner-3-exon4
TP53-control

Query  3     CCGTGCCGGCCCTAGCGATGGATGATTTGTATGCTGTCCCCGG-CGATATTGAACAATGG  61
             || ||||| ||| ||| |||||||||||| ||||||||||||| ||||||||||||||||
Sbjct  7     CCTTGCCGTCCCAAGCAATGGATGATTTG-ATGCTGTCCCCGGACGATATTGAACAATGG  65

Query  62    TTCACTGAAGACCCAGGTCCAGATGAAGCTCCCAGAATGCCAGAGG-TGTCCTCCCCCCG  120
             |||||||||||||||||||||||||||||||||||||||||||||| ||  |||||||||
Sbjct  66    TTCACTGAAGACCCAGGTCCAGATGAAGCTCCCAGAATGCCAGAGGCTG--CTCCCCCCG  123

Query  121   TGCCCCCTGCACCCAGCGCCTCCTACACCCGGGCGGCCCCCTGGCCCCCAGGCCCCTCCT  180
             || |||||||||| |||  |||||||||| || ||||||| || | || || ||||||||
Sbjct  124   TGGCCCCTGCACC-AGCAGCTCCTACACC-GG-CGGCCCC-TG-CACC-AGCCCCCTCCT  177
```

```
Turner-4-exon4
TP53-control

Query  1     CCCTTG-CG-CTCATGC-ATGGATGATTTGATGCTGTCCCCGG-CGATATTGAACAATGG  56
             |||||| || | || || ||||||||||||||||||||||||| ||||||||||||||||
Sbjct  6     CCCTTGCCGTCCCAAGCAATGGATGATTTGATGCTGTCCCCGGACGATATTGAACAATGG  65

Query  57    -TCACTGAAGACCCAGGTCCAGATGAAGCTCCCAGAATGCCAGAGGCTGCTCCCCCCGTG  115
              |||||||||||||||||||||||||||||||||||||||||||||||||||||||||||
Sbjct  66    TTCACTGAAGACCCAGGTCCAGATGAAGCTCCCAGAATGCCAGAGGCTGCTCCCCCCGTG  125

Query  116   GCCC-T-CACCAGCGGCT-CTACACCGGCGGCCCCT-CACCAGCCCCCTTCTGGCCCCTG  171
             |||| | ||||||| ||| ||||||||||||||||| |||||||||||| ||||||||||
Sbjct  126   GCCCCTGCACCAGCAGCTCCTACACCGGCGGCCCCTGCACCAGCCCCCTCCTGGCCCCTG  185

Query  172   T-A-CTTCTGTCC--TCCCAGAAAACCTACCAGG-CAGCTACGGTT-CCGTCTGG-CTTC  224
             | | |||||||||  ||||||||||||||||||| ||||||||||| |||||||| ||||
Sbjct  186   TCATCTTCTGTCCCTTCCCAGAAAACCTACCAGGGCAGCTACGGTTTCCGTCTGGGCTTC  245

Query  225   TT-CAT-C-GGGACAGCCAAGCCTGTGA-TTGCAACGG  258
             || ||| | |||||||||||| |||||| ||||| |||
Sbjct  246   TTGCATTCTGGGACAGCCAAGTCTGTGACTTGCA-CGG  282
```

```
Turner-6-exon4
TP53-control

Query  18    AGTCCTTCTTGCCGTCCCA--CAATGGATGATTTGATGCTGTCCCCGG-C-ATATTGAAC  73
             |||||  ||||||||||||  ||||||||||||||||||||||||||| | |||||||||
Sbjct  1     AGTCCCCCTTGCCGTCCCAAGCAATGGATGATTTGATGCTGTCCCCGGACGATATTGAAC  60

Query  74    AATGGTTCACTGAAGACCCAGGTCCAGATGAAGCTCCCAGAAT-CCAG-GGCTGCT-CCC  130
             ||||||||||||||||||||||||||||||||||||||||||| |||| ||||||| |||
Sbjct  61    AATGGTTCACTGAAGACCCAGGTCCAGATGAAGCTCCCAGAATGCCAGAGGCTGCTCCCC  120
```

```
Query  131  CCGTGG-CCCTG-ACCAGCAGCTCCTACACCGGCGGCCC-TG-ACCAG-CCCCTCCT-GC  184
            |||||| ||||| |||||||||||||||||||||||||| || ||||| |||||||| ||
Sbjct  121  CCGTGGCCCCTGCACCAGCAGCTCCTACACCGGCGGCCCCTGCACCAGCCCCCTCCTGGC  180

Query  185  CCCTGTCATCTTCTGTCCCTTCCCAG-AAACCTACCAGGGCAGCTACGGTTTCCGTCTGG  243
            |||||||||||||||||||||||||| |||||||||||||||||||||||||||||||||
Sbjct  181  CCCTGTCATCTTCTGTCCCTTCCCAGAAAACCTACCAGGGCAGCTACGGTTTCCGTCTGG  240

Query  244  -CTTCT-G-ATTCTGGG  257
             ||||| | ||||||||
Sbjct  241  GCTTCTTGCATTCTGGG  257
```

Turner-11-exon4
TP53-control

```
Query  16   ATGGATGATTTGATGCTGTCCCCGG-CGATATTGAACAATGGTTCACTGAAGACCCAGGT  74
            ||||||||||||||||||||||||| ||||||||||||||||||||||||||||||||||
Sbjct  24   ATGGATGATTTGATGCTGTCCCCGGACGATATTGAACAATGGTTCACTGAAGACCCAGGT  83

Query  75   CCAGATGAAGCTCC-AGA-TGCCAGAGCGTGCCCCCCGCGTGGCCCCTGCACCAGCAGCT  132
            |||||||||||||| ||| ||||||||  ||| |||| ||||||||||||||||||||||
Sbjct  84   CCAGATGAAGCTCCCAGAATGCCAGAGGCTGCTCCCCCCGTGGCCCCTGCACCAGCAGCT  143

Query  133  CCTACACCGGCGGCCCCTGC-CCAGCCCCCTCCTGGGCCCCTG-CATCTTCTGGTCCTTC  190
            |||||||||||||||||||| ||||||||||||||| |||||| |||||||||  |||||
Sbjct  144  CCTACACCGGCGGCCCCTGCACCAGCCCCCTCCTGG-CCCCTGTCATCTTCTGTCCCTTC  202

Query  191  CCAGAAAAC-TACCAGG-CAGCTACGG-TTCCGGCTGGGCTTATTGCATTG-GGGACA-C  245
            ||||||||| ||||||| ||||||||| ||||| |||||||| |||||||  |||||| |
Sbjct  203  CCAGAAAACCTACCAGGGCAGCTACGGTTTCCGTCTGGGCTTCTTGCATTCTGGGACAGC  262

Query  246  CAAG-CTGTGA  255
            |||| ||||||
Sbjct  263  CAAGTCTGTGA  273
```
