# Supplementary material for: Genomic instability in individuals with sex determination defects and germ cell cancer
Source: Cell Death Discov. 2023 May 23;9:173. doi: 10.1038/s41420-023-01470-6 (PMC10202957; doi:10.1038/s41420-023-01470-6)

Some of the membranes were cur and the pieces were processed separately with different antibodies. This allowed us to analyze different markers from the same gel and also save the material.

Figure 1a

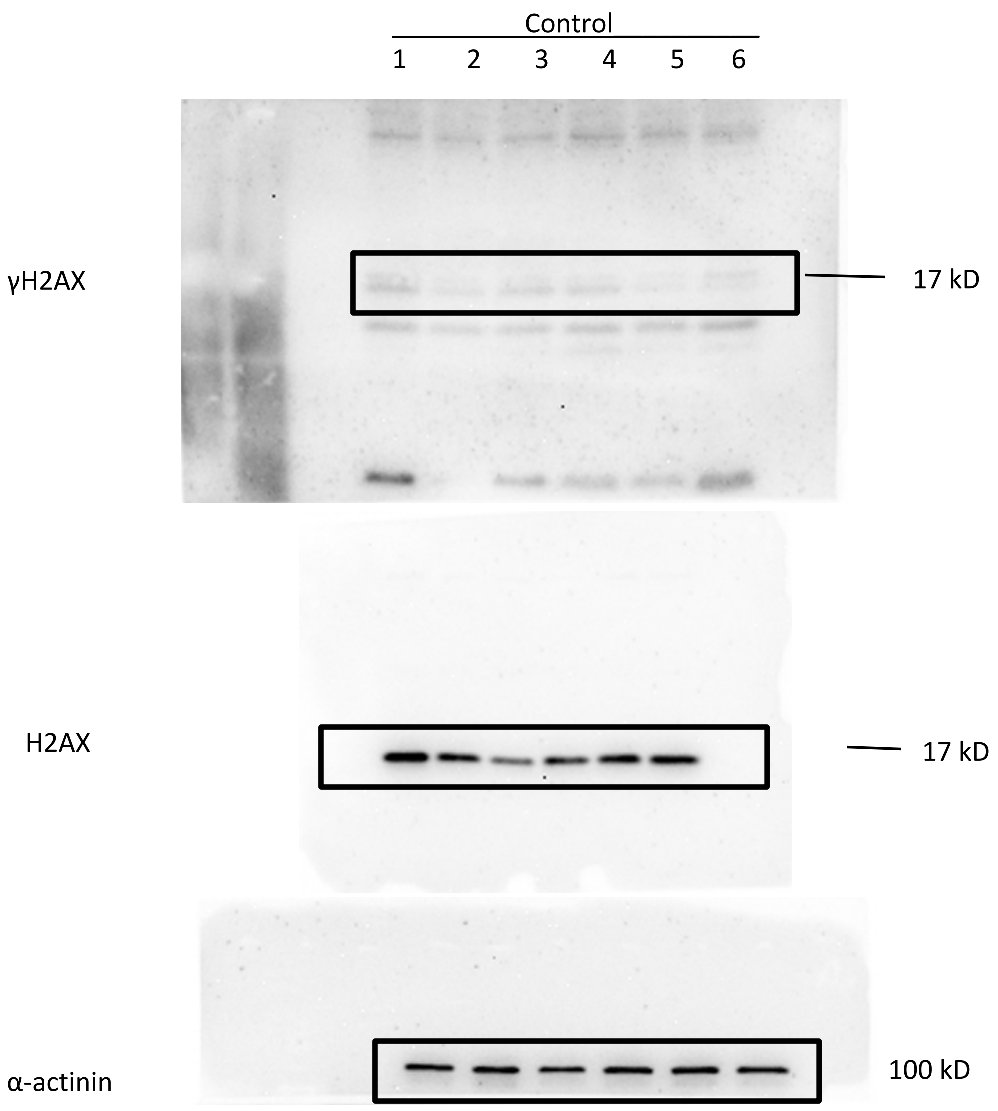

Figure 1b

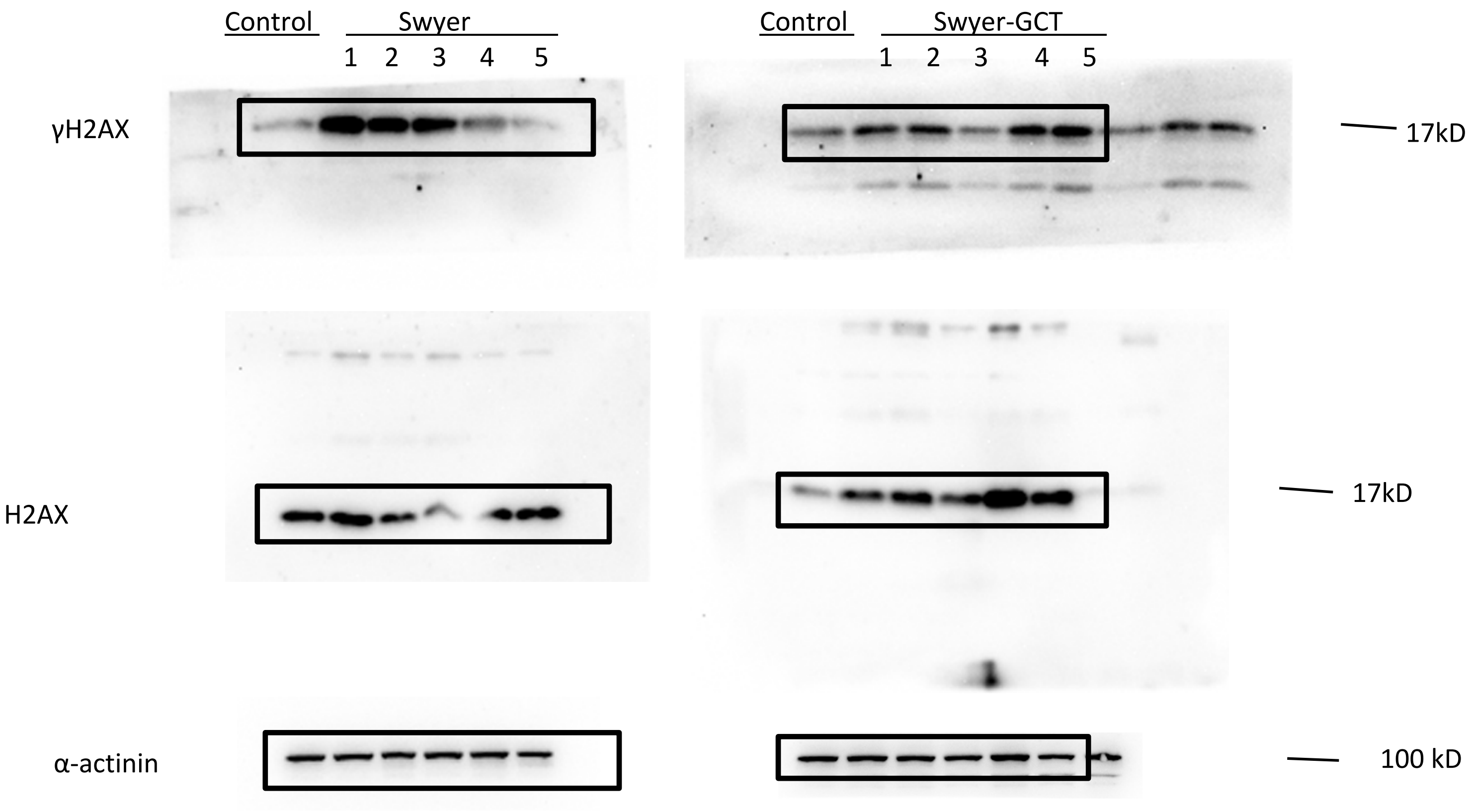
Control
Swyer
1 2 3 4 5
Control
Swyer-GCT
1 2 3 4 5
γH2AX
17kD
H2AX
17kD
α-actinin
100 kD

Figure 1d

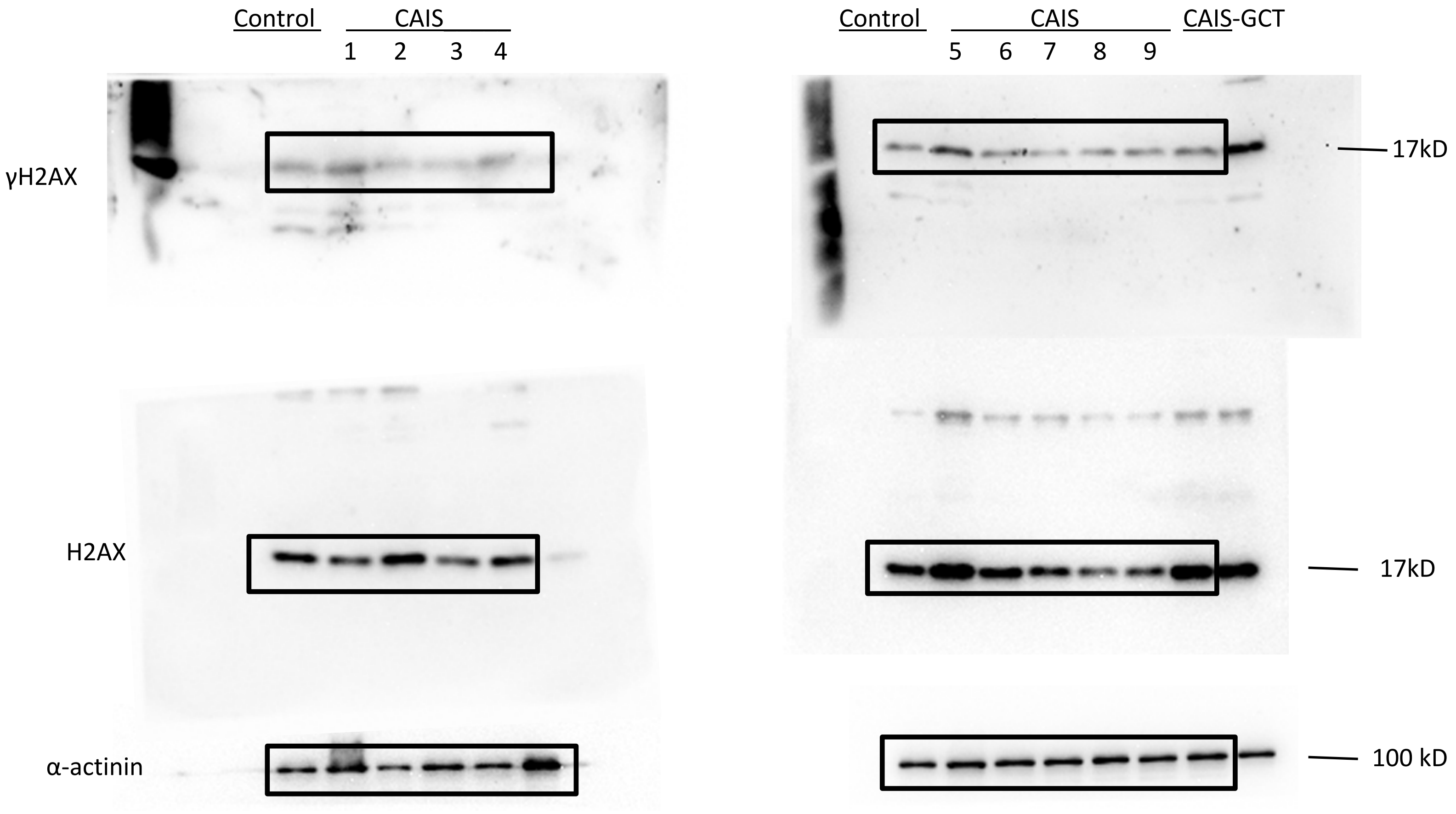

Control
CAIS
1
2
3
4
γH2AX
H2AX
α-actinin
Control
CAIS
CAIS-GCT
5
6
7
8
9
17kD
17kD
100 kD

Figure 1e

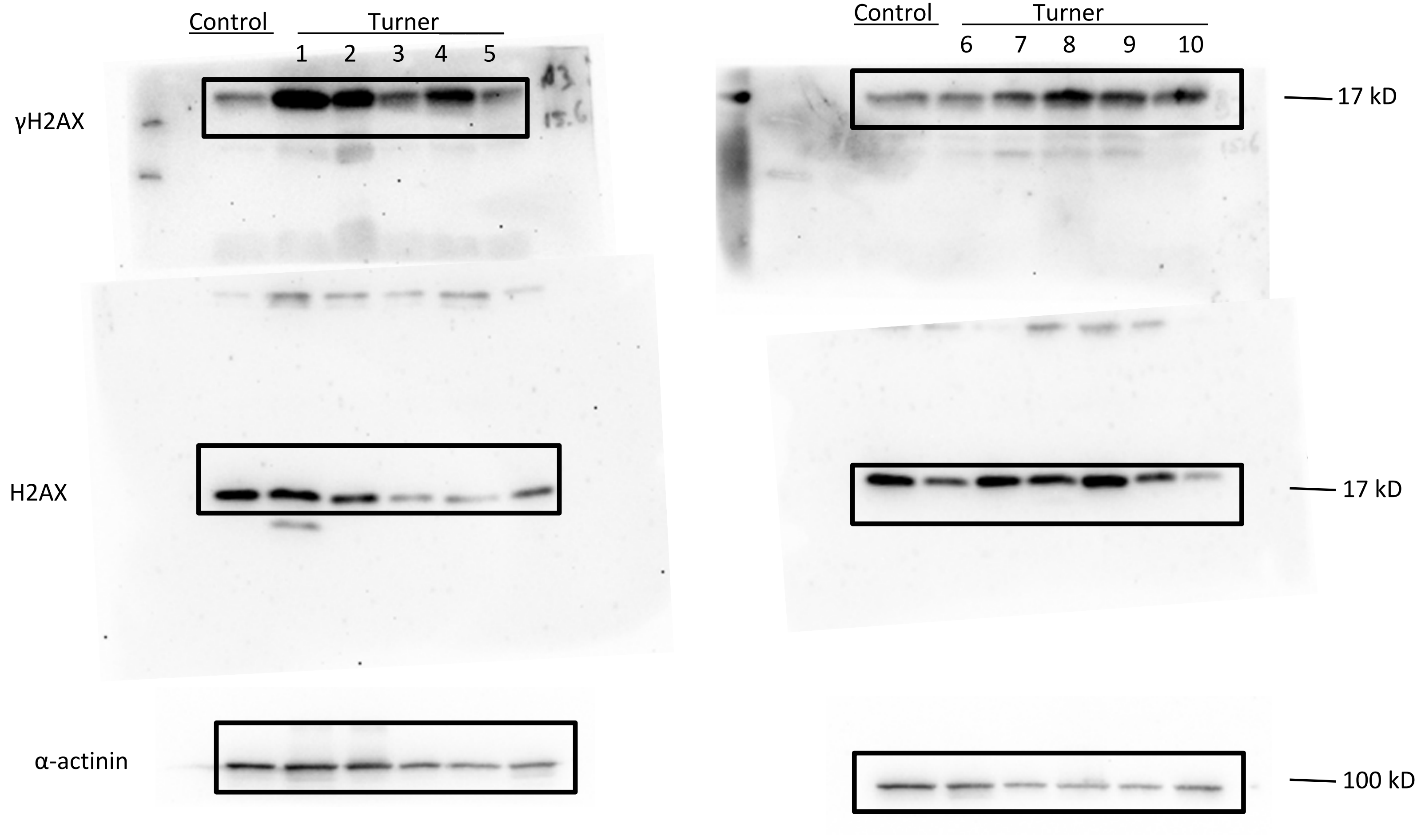

Control
Turner
1
2
3
4
5
γH2AX
H2AX
α-actinin
Control
Turner
6
7
8
9
10
17 kD
17 kD
100 kD

Figure 1f

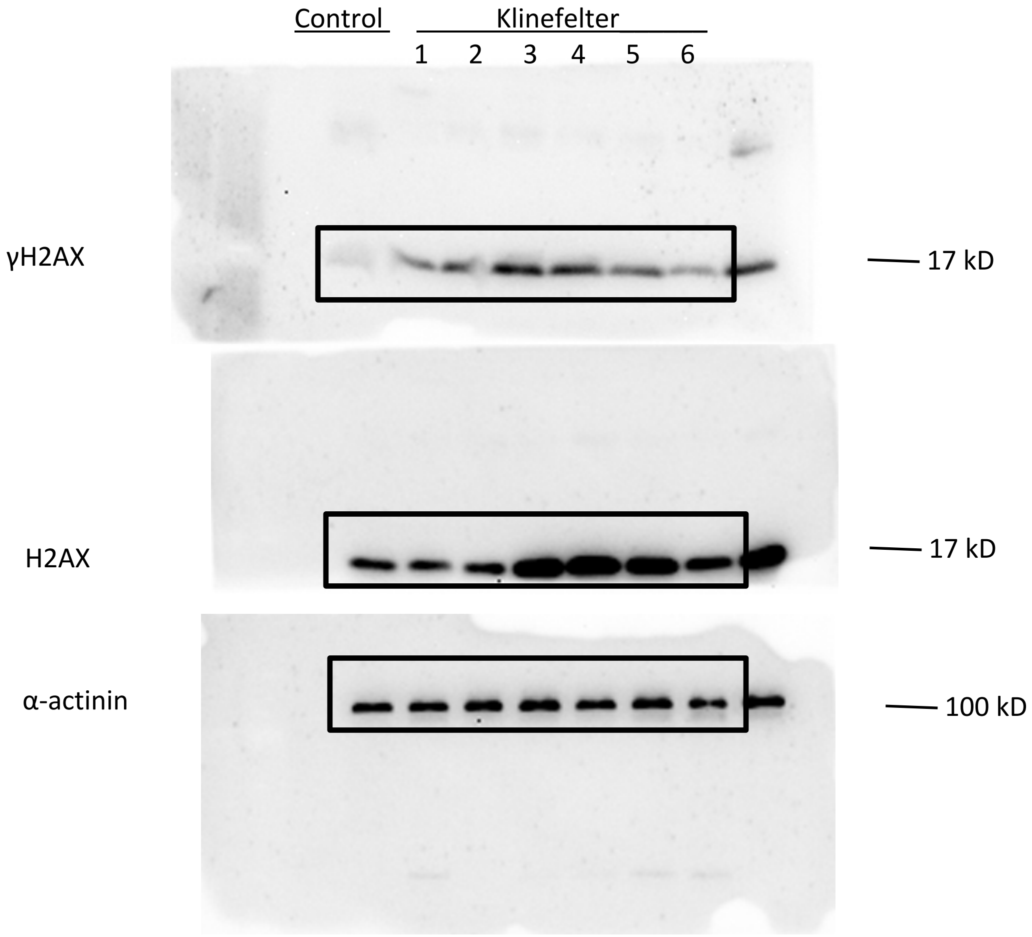
Control
Klinefelter
1
2
3
4
5
6
γH2AX
17 kD
H2AX
17 kD
α-actinin
100 kD

Figure 1i

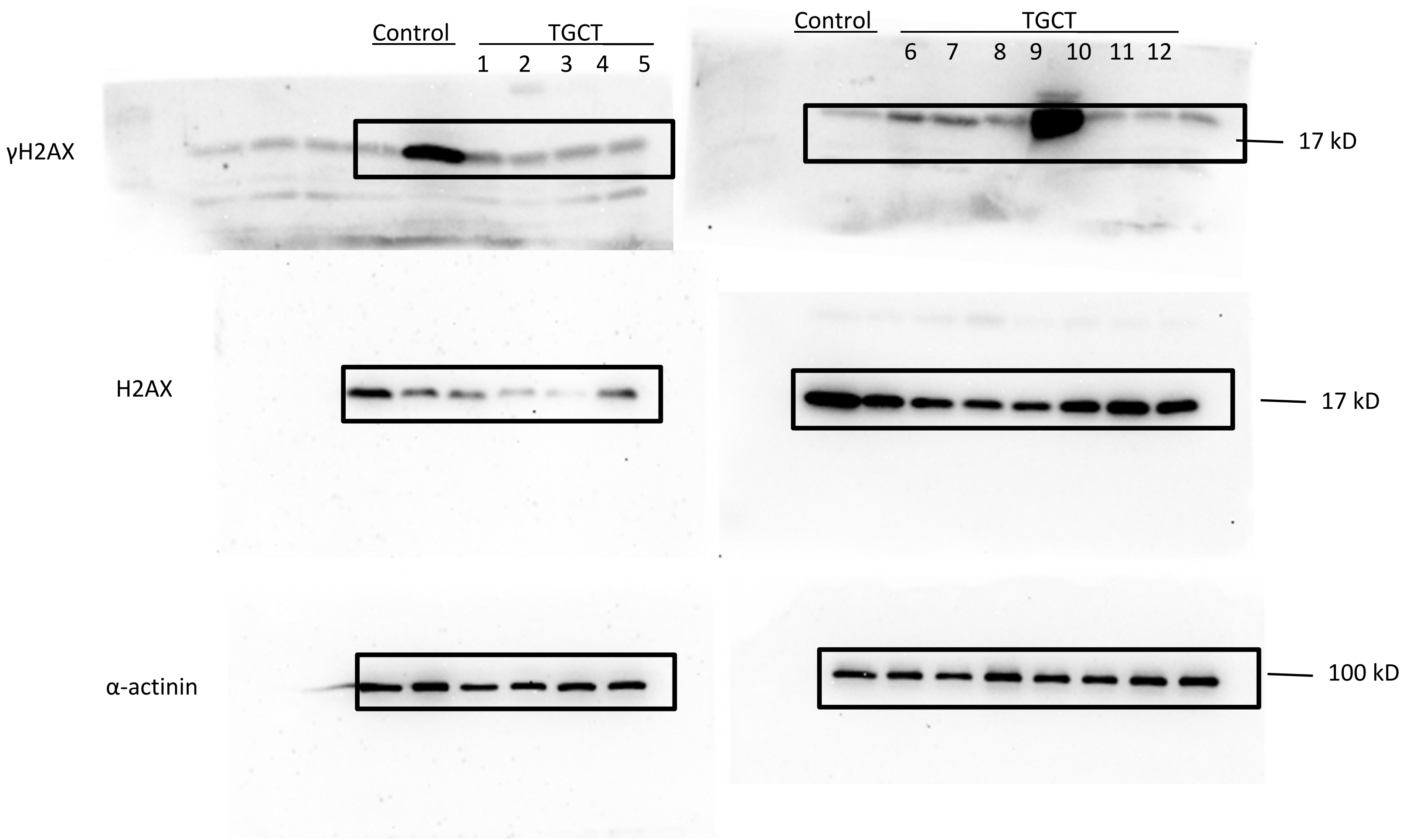
Control
TGCT
1 2 3 4 5
Control
TGCT
6 7 8 9 10 11 12
γH2AX
17 kD
H2AX
17 kD
α-actinin
100 kD

Figure 4f

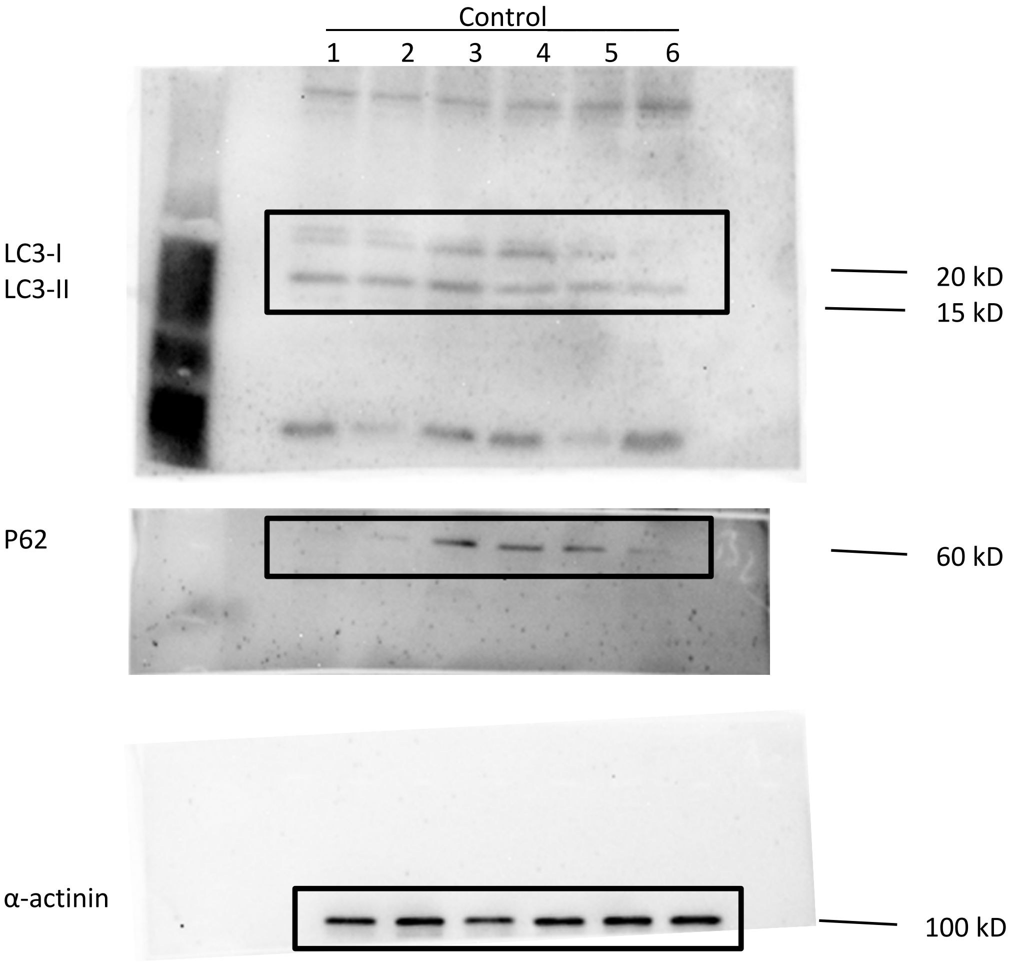
Control
1
2
3
4
5
6
LC3-I
LC3-II
20 kD
15 kD
P62
60 kD
α-actinin
100 kD

Figure 4g

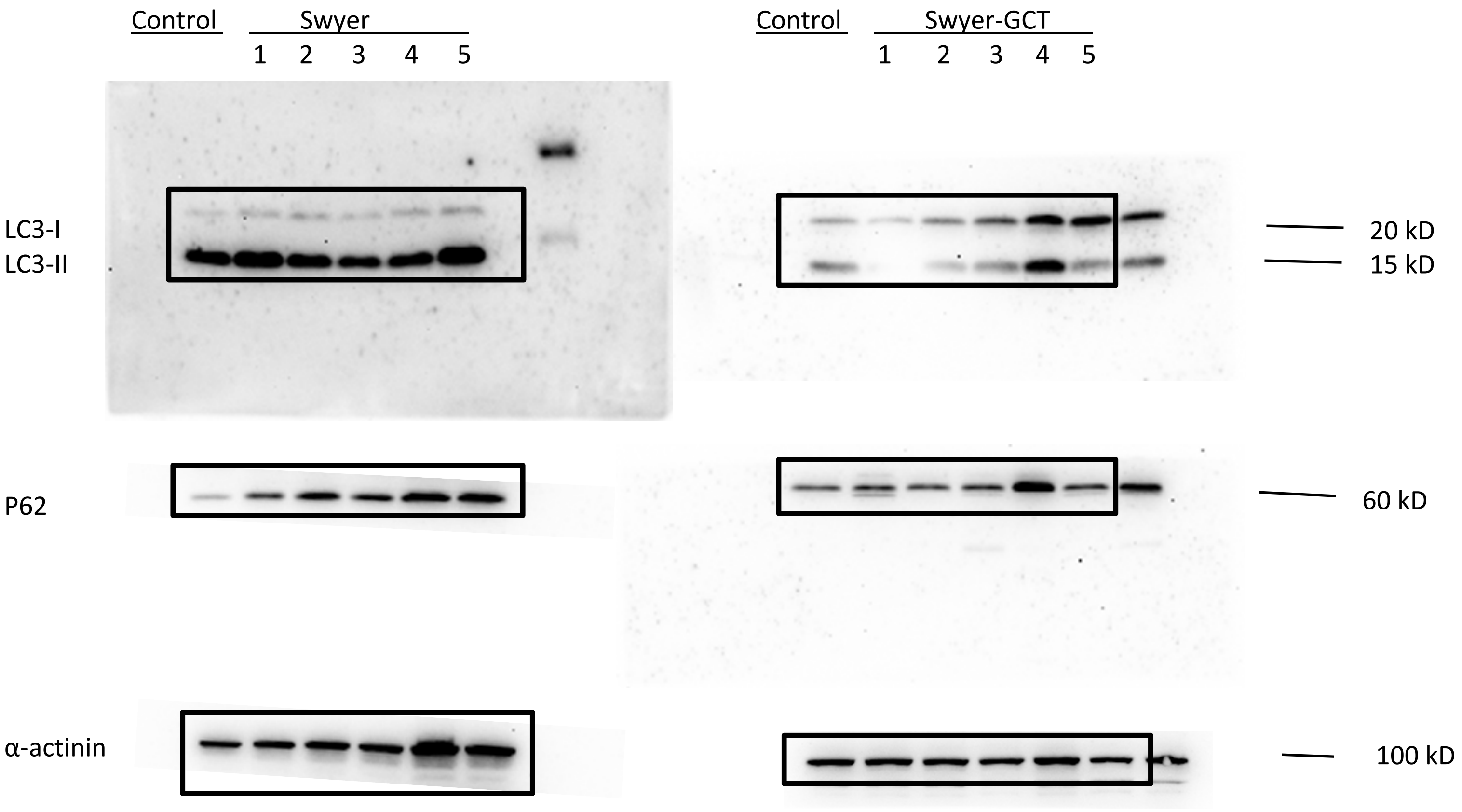
Control
Swyer
1 2 3 4 5
Control
Swyer-GCT
1 2 3 4 5
LC3-I
LC3-II
P62
α-actinin
20 kD
15 kD
60 kD
100 kD

Figure 4h

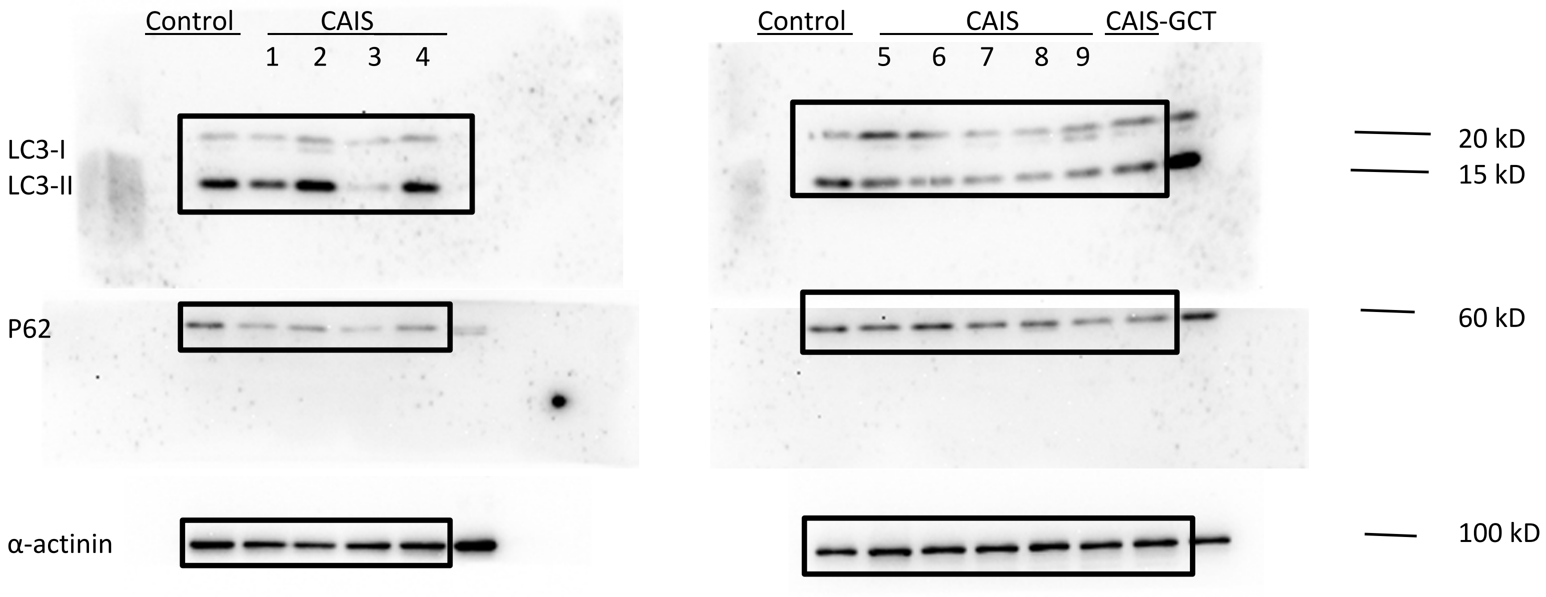

Control
CAIS
1 2 3 4
LC3-I
LC3-II
P62
α-actinin
Control
CAIS
CAIS-GCT
5 6 7 8 9
20 kD
15 kD
60 kD
100 kD

Figure 4i

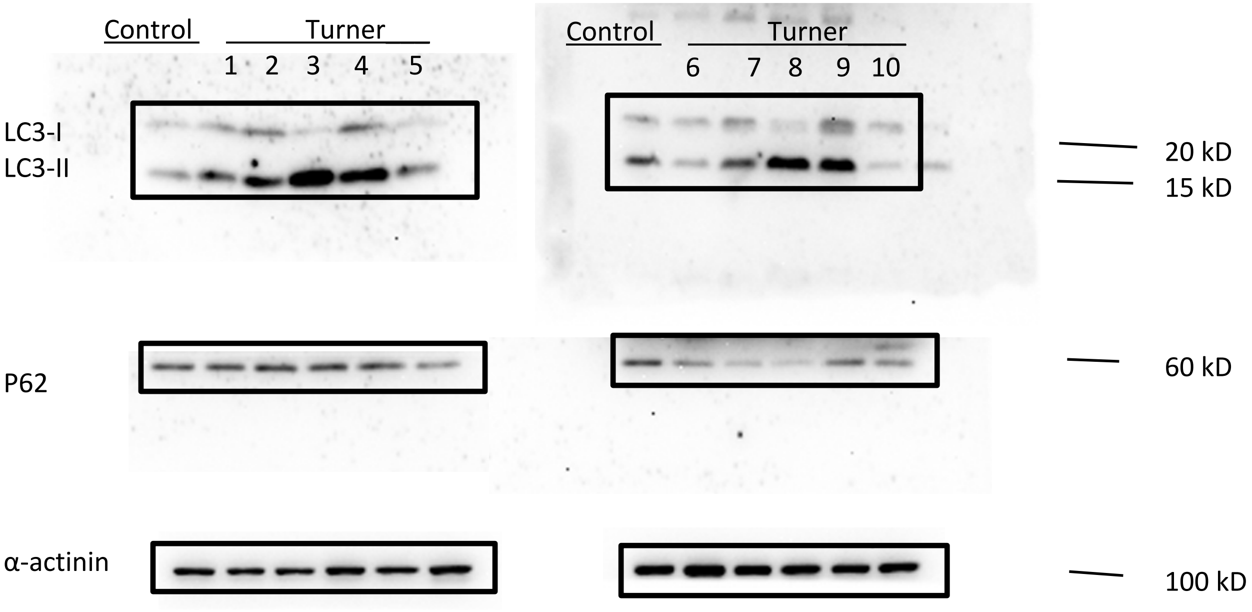

Control
Turner
1 2 3 4 5
Control
Turner
6 7 8 9 10
LC3-I
LC3-II
P62
α-actinin
20 kD
15 kD
60 kD
100 kD

Figure 4j

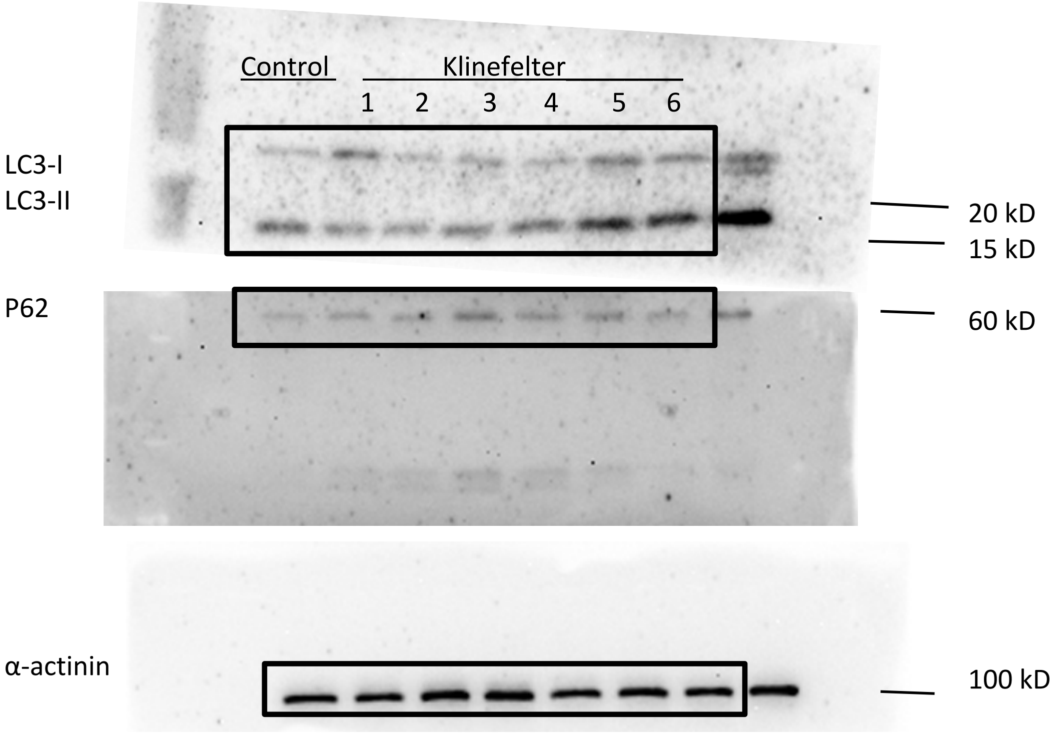

Control
Klinefelter
1 2 3 4 5 6
LC3-I
LC3-II
20 kD
15 kD
P62
60 kD
α-actinin
100 kD

Figure 4m

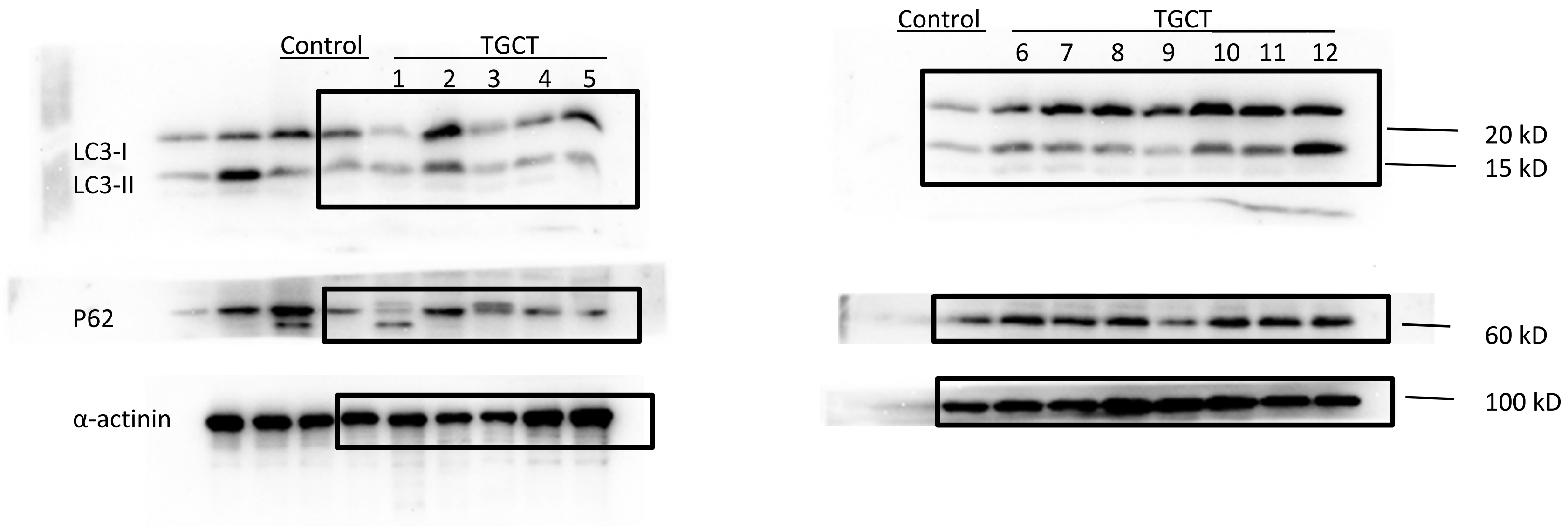

Control
TGCT
1 2 3 4 5
LC3-I
LC3-II
P62
α-actinin
Control
TGCT
6 7 8 9 10 11 12
20 kD
15 kD
60 kD
100 kD

Figure 5a

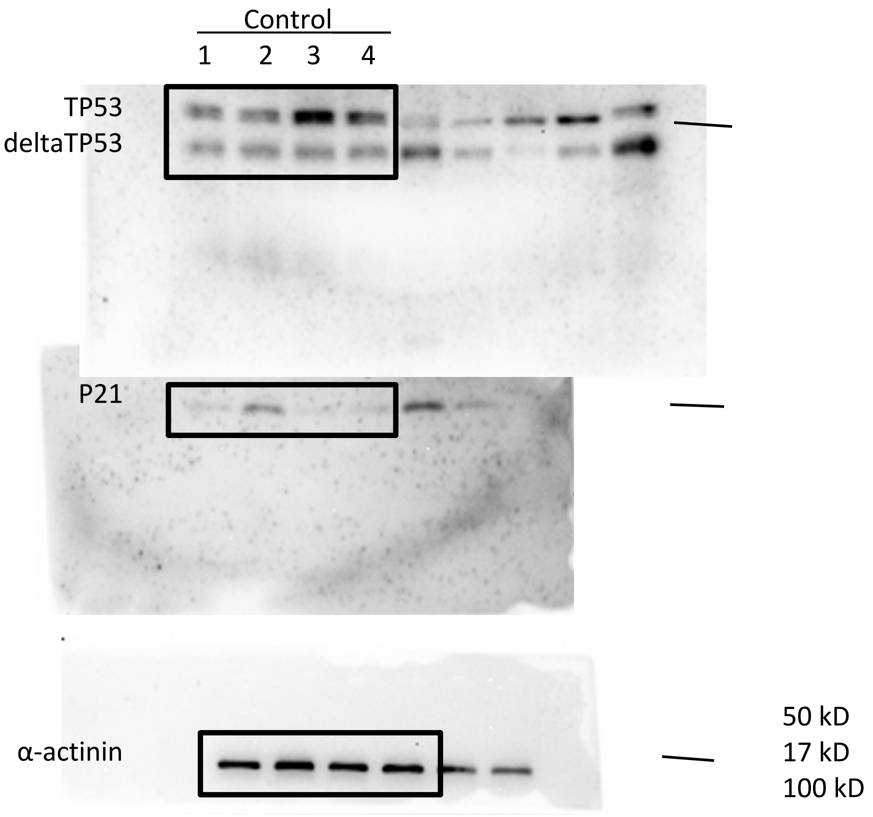

Figure 5b

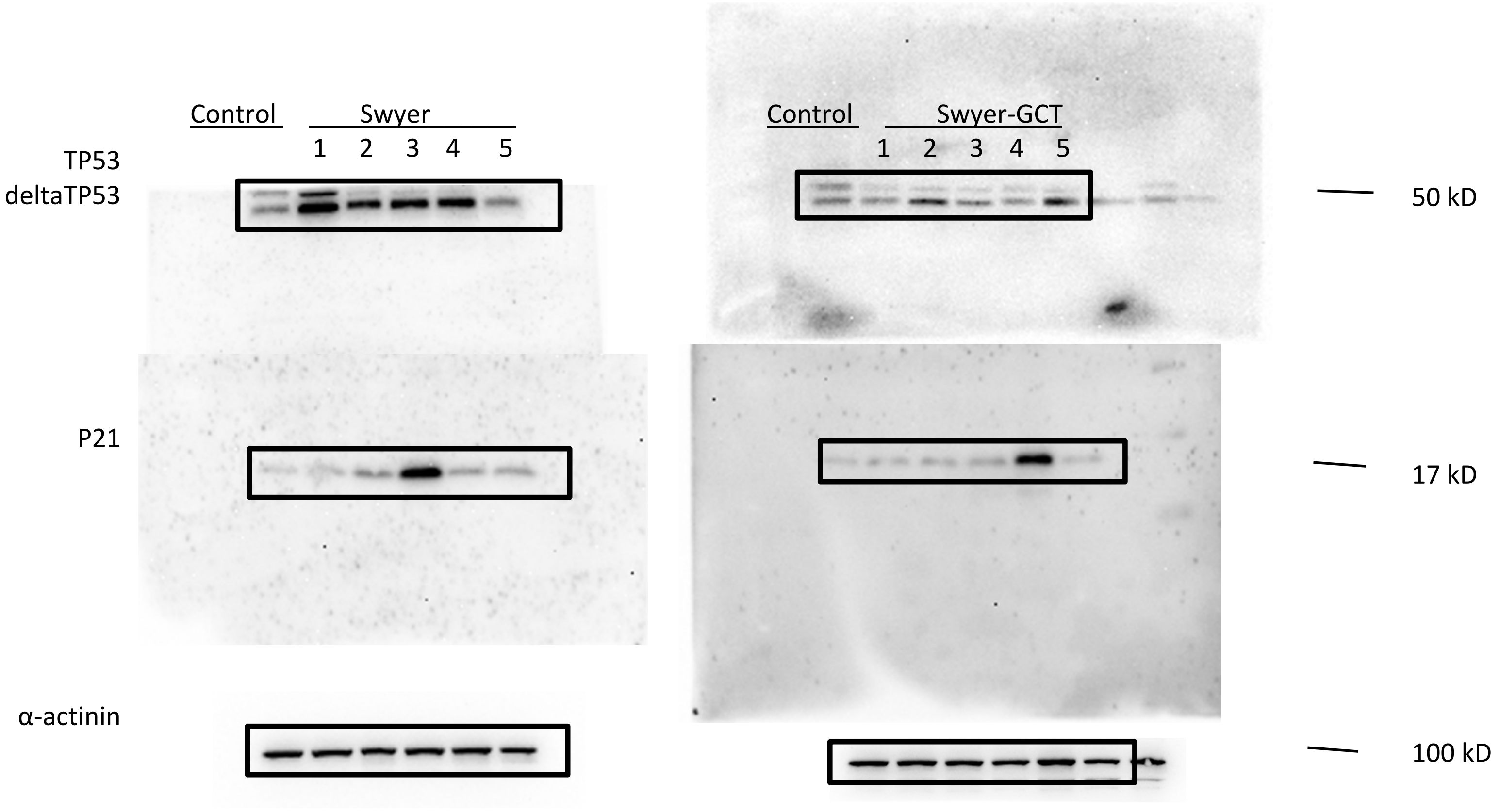
Control
Swyer
1 2 3 4 5
TP53
deltaTP53
P21
α-actinin
Control
Swyer-GCT
1 2 3 4 5
50 kD
17 kD
100 kD

Figure 5c

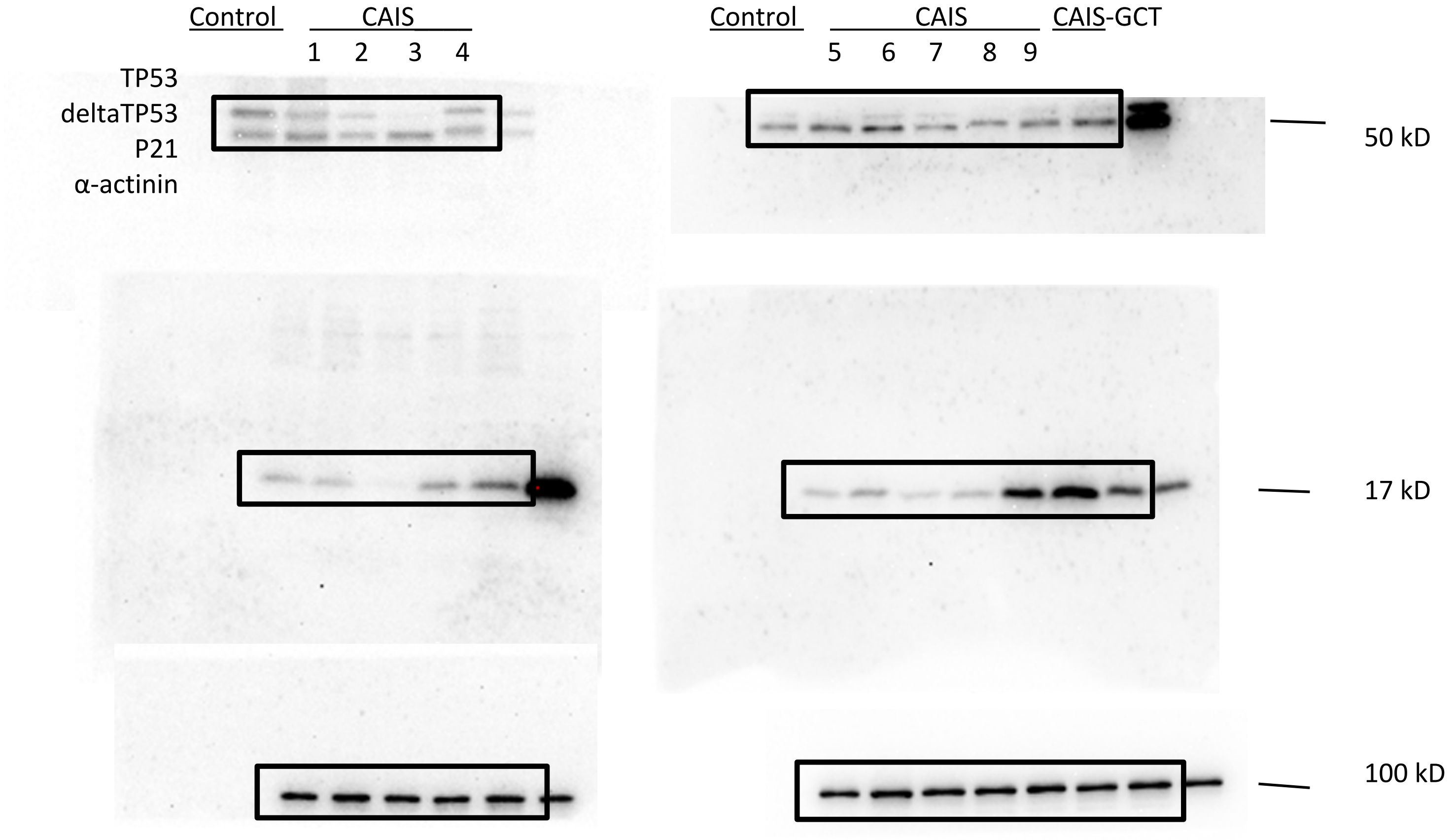

Control
CAIS
1
2
3
4
TP53
deltaTP53
P21
α-actinin
Control
CAIS
CAIS-GCT
5
6
7
8
9
50 kD
17 kD
100 kD

Figure 5d

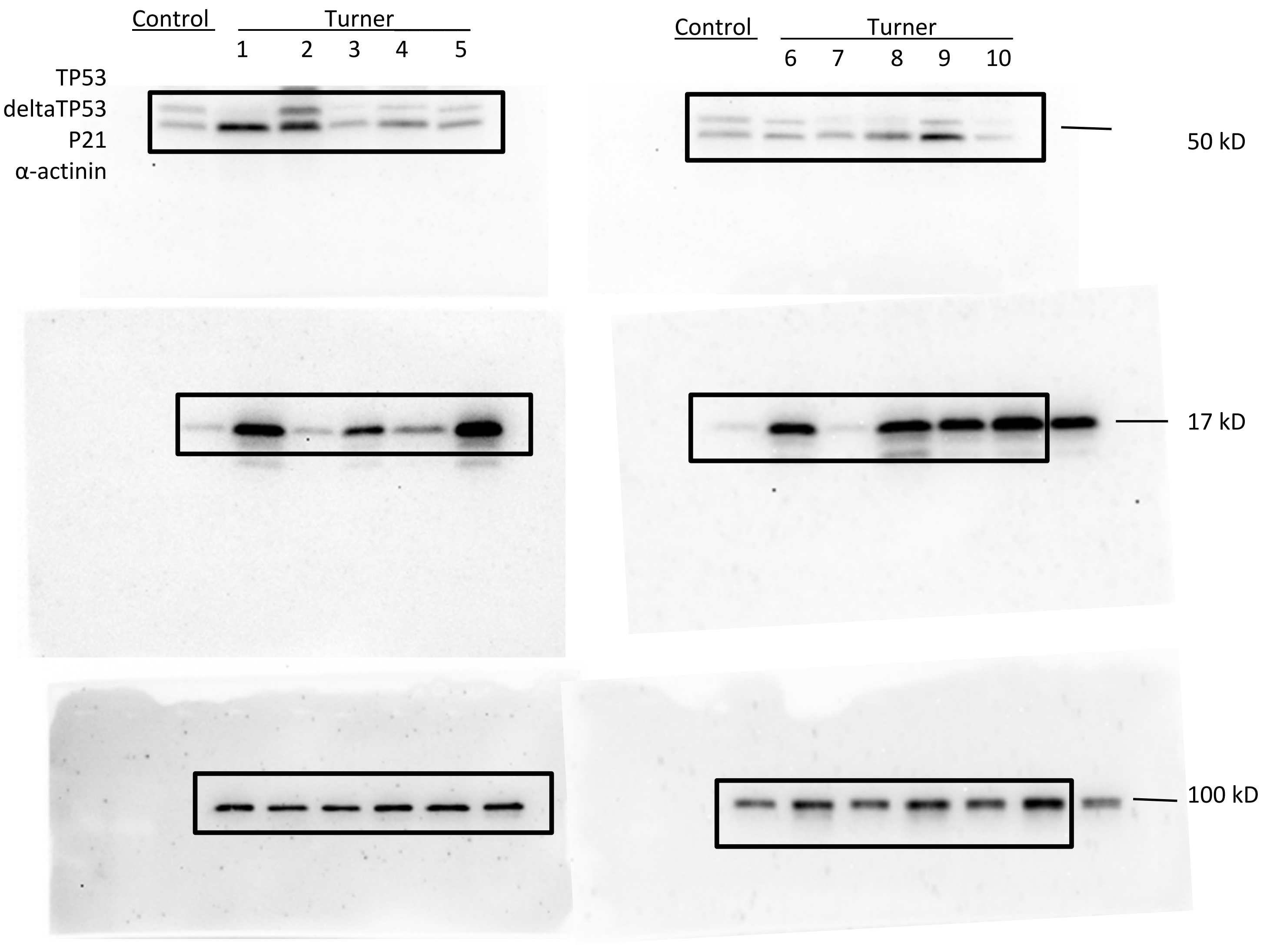

Control
Turner
1
2
3
4
5
TP53
deltaTP53
P21
α-actinin
Control
Turner
6
7
8
9
10
50 kD
17 kD
100 kD

Figure 5e

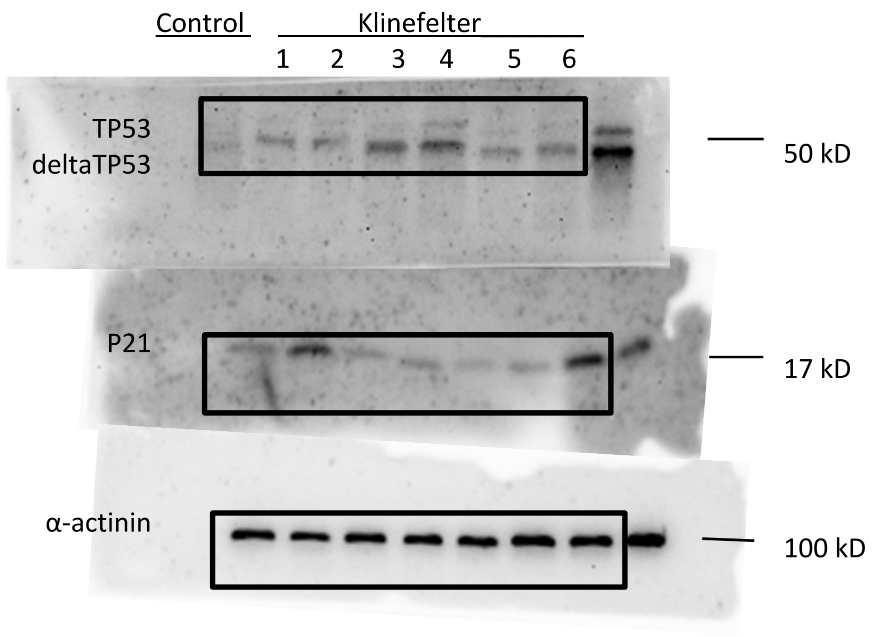
Control
Klinefelter
1 2 3 4 5 6
TP53
deltaTP53
50 kD
P21
17 kD
α-actinin
100 kD

Figure 5g

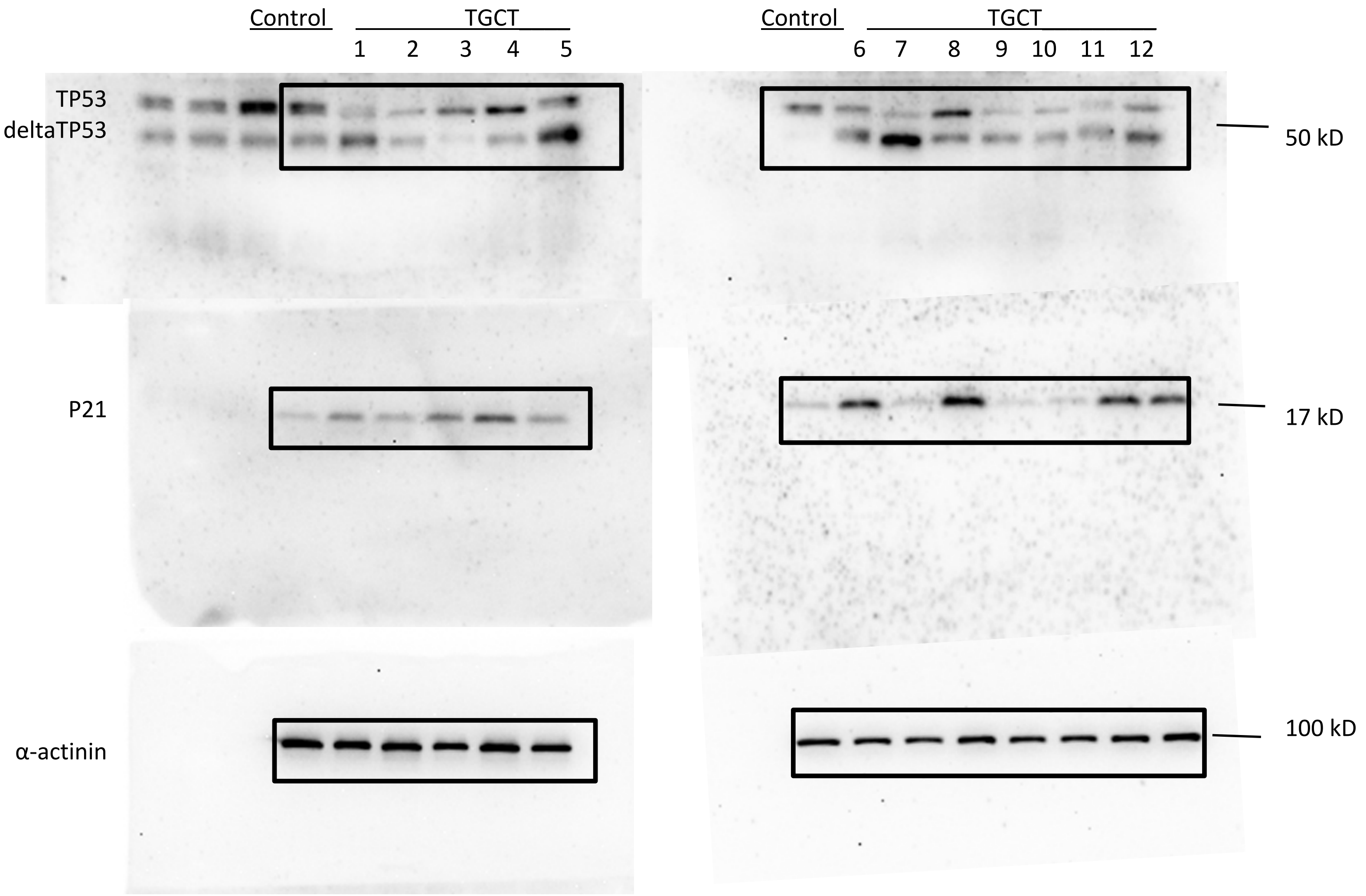

Control
TGCT
1
2
3
4
5
Control
TGCT
6
7
8
9
10
11
12
TP53
deltaTP53
50 kD
P21
17 kD
α-actinin
100 kD

Figure 7a

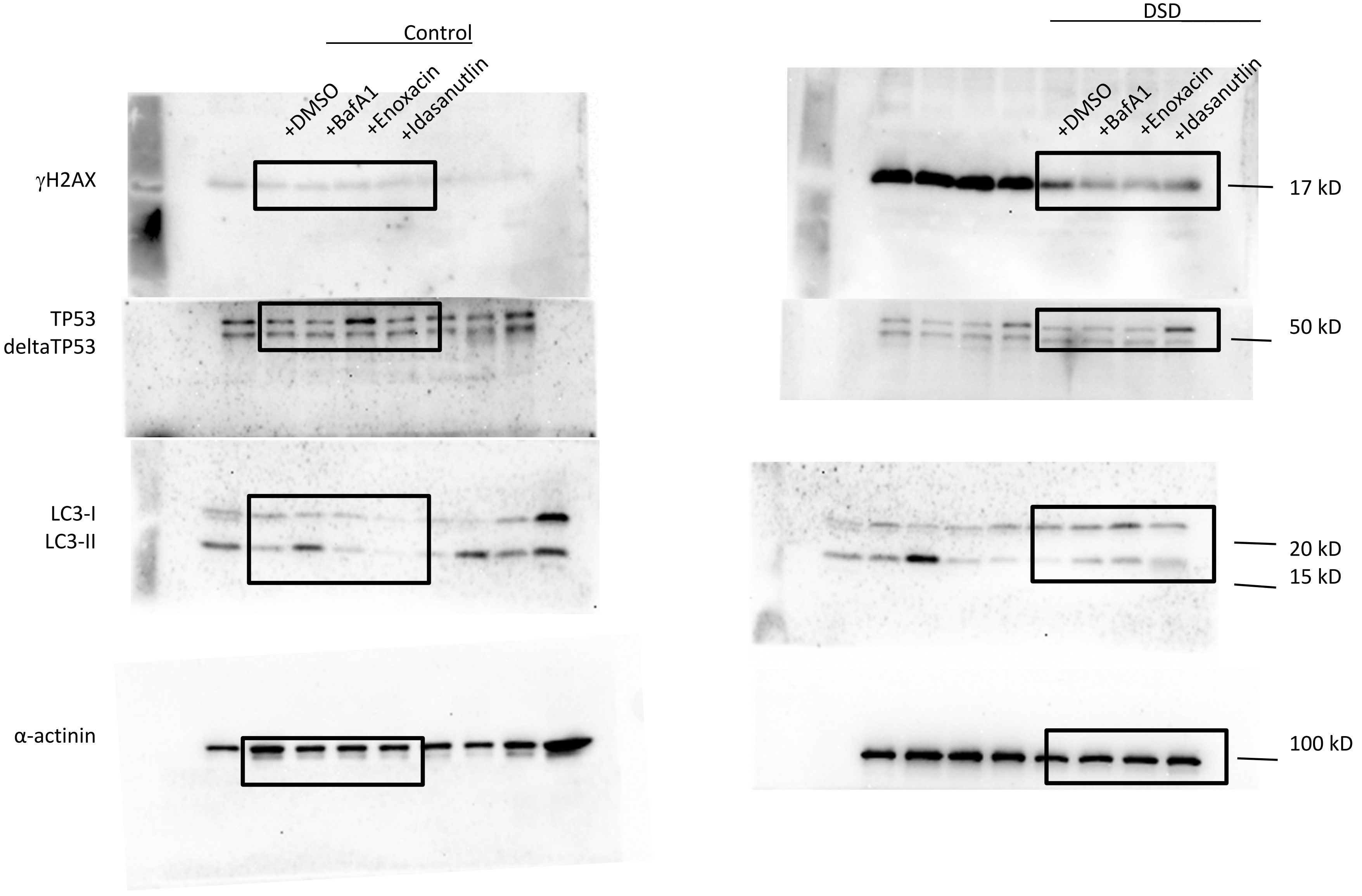
Control
DSD
+DMSO
+BafA1
+Enoxacin
+Idasanutlin
γH2AX
TP53
deltaTP53
LC3-I
LC3-II
α-actinin
17 kD
50 kD
20 kD
15 kD
100 kD

Figure 7e

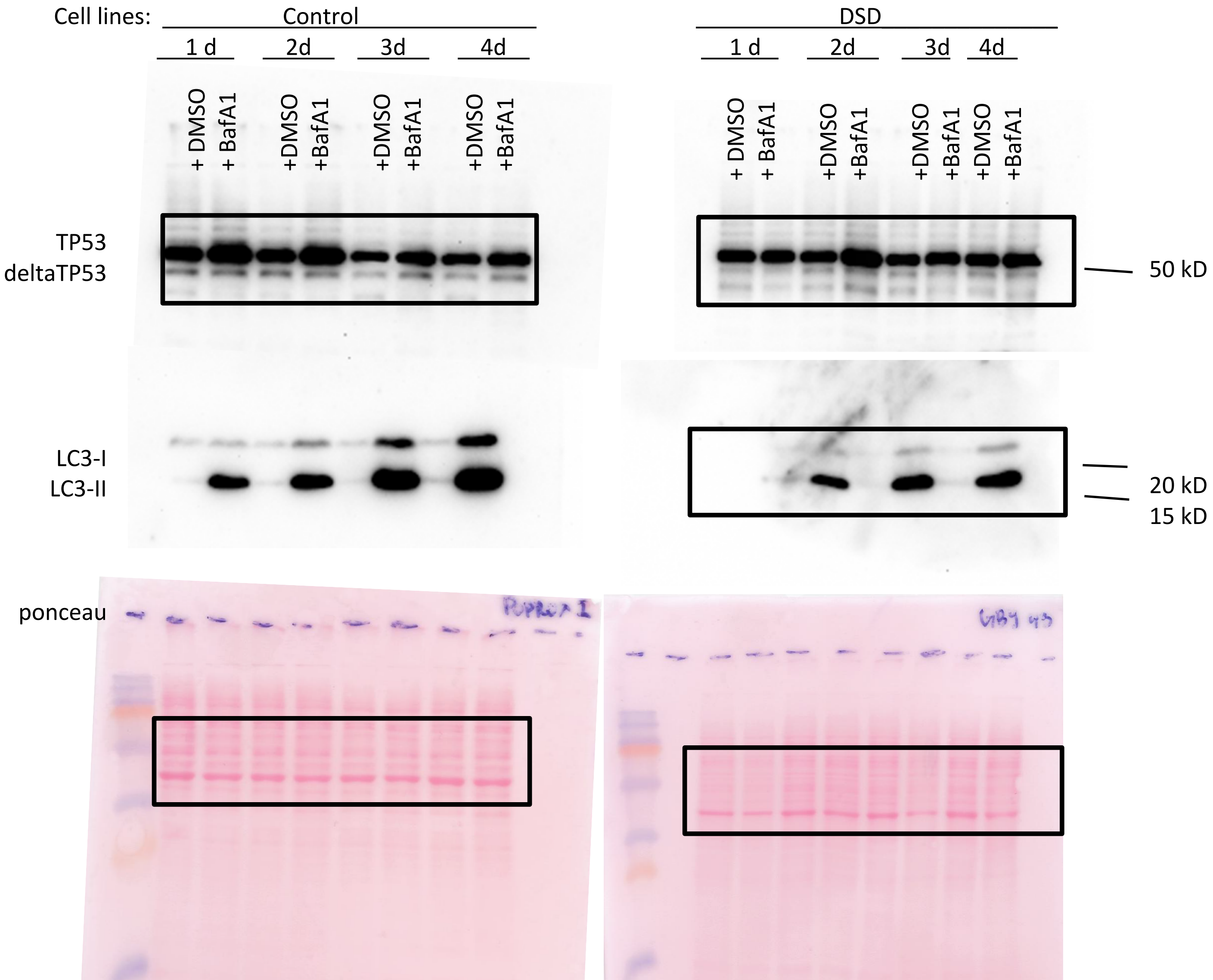
Cell lines:
Control
DSD
1 d
2d
3d
4d
+ DMSO
+ BafA1
+DMSO
+BafA1
TP53
deltaTP53
50 kD
LC3-I
LC3-II
20 kD
15 kD
ponceau

Extended data figure 3e

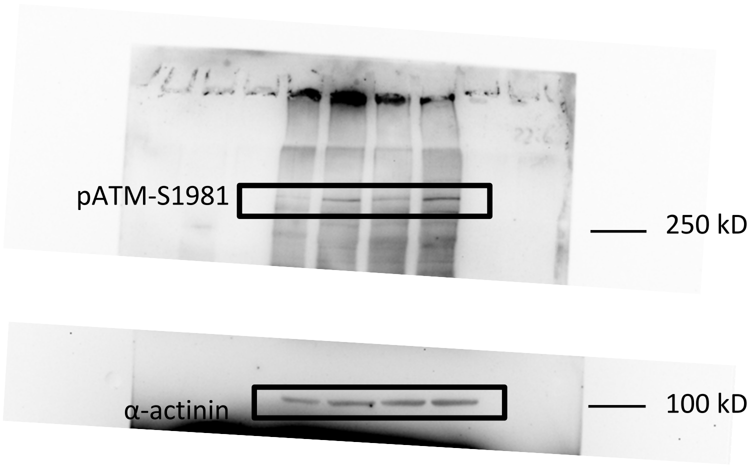

Supplement: Supplementary file 15 — Original Data File [file 41420_2023_1470_MOESM15_ESM.pdf]
